# Using a chat-based informed consent tool in large-scale genomic research

**DOI:** 10.1093/jamia/ocad181

**Published:** 2023-09-04

**Authors:** Sarah K Savage, Jonathan LoTempio, Erica D Smith, E Hallie Andrew, Gloria Mas, Amanda H Kahn-Kirby, Emmanuèle Délot, Andrea J Cohen, Georgia Pitsava, Robert Nussbaum, Vincent A Fusaro, Seth Berger, Eric Vilain

**Affiliations:** Invitae Corporation, San Francisco, CA, United States; Institute for Clinical and Translational Science, University of California, Irvine, CA, United States; Invitae Corporation, San Francisco, CA, United States; Division of Genetics and Metabolism, Children's National Rare Disease Institute, Washington, DC, United States; Center for Genetic Medicine Research, Children's National Research Institute, Washington, DC, United States; Invitae Corporation, San Francisco, CA, United States; Invitae Corporation, San Francisco, CA, United States; Institute for Clinical and Translational Science, University of California, Irvine, CA, United States; Center for Genetic Medicine Research, Children's National Research Institute, Washington, DC, United States; Department of Genomics and Precision Medicine, George Washington University, Washington, DC, United States; Center for Genetic Medicine Research, Children's National Research Institute, Washington, DC, United States; Center for Genetic Medicine Research, Children's National Research Institute, Washington, DC, United States; Invitae Corporation, San Francisco, CA, United States; Invitae Corporation, San Francisco, CA, United States; Institute for Clinical and Translational Science, University of California, Irvine, CA, United States; Division of Genetics and Metabolism, Children's National Rare Disease Institute, Washington, DC, United States; Center for Genetic Medicine Research, Children's National Research Institute, Washington, DC, United States; Department of Genomics and Precision Medicine, George Washington University, Washington, DC, United States; Institute for Clinical and Translational Science, University of California, Irvine, CA, United States; Center for Genetic Medicine Research, Children's National Research Institute, Washington, DC, United States; Department of Genomics and Precision Medicine, George Washington University, Washington, DC, United States

**Keywords:** informed consent, chatbot, genomics, genetic counseling, large-scale research

## Abstract

**Objective:**

We implemented a chatbot consent tool to shift the time burden from study staff in support of a national genomics research study.

**Materials and Methods:**

We created an Institutional Review Board-approved script for automated chat-based consent. We compared data from prospective participants who used the tool or had traditional consent conversations with study staff.

**Results:**

Chat-based consent, completed on a user’s schedule, was shorter than the traditional conversation. This did not lead to a significant change in affirmative consents. Within affirmative consents and declines, more prospective participants completed the chat-based process. A quiz to assess chat-based consent user understanding had a high pass rate with no reported negative experiences.

**Conclusion:**

Our report shows that a structured script can convey important information while realizing the benefits of automation and burden shifting. Analysis suggests that it may be advantageous to use chatbots to scale this rate-limiting step in large research projects.

## Background and significance

Traditional informed consent is obtained via direct interaction between a member of a study team and the individual considering enrollment or their legally authorized representative or guardian, either in person or via phone/video call.^1–3^

This requires that the:

1. individual has geographic proximity or technological access to the study staff;
2. patient and study personnel have the available time and resources to meet; and
3. study team reviews consent materials fully with prospective participants.

Large-scale projects can include thousands of participants, with far fewer staff members with limited availability. This creates a bottleneck where staff working hours and patient availability limits the number of participants who can be brought into a study.^4^^,^^5^ At the start of the new large-scale NHGRI-funded Genomics Research to Elucidate the Genetics of Rare Diseases (GREGoR) Consortium, we implemented a chat-based consent to alleviate this bottleneck of traditional consent within the Pediatric Mendelian Genomic Research Center (PMGRC).

The PMGRC is a collaboration between Children’s National Hospital (CNH), University of California, Irvine, and Invitae, and is 1 of 5 centers in the GREGoR Consortium. GREGoR’s goal is to discover the cause of currently unexplained genetic phenotypes.^6^ To accomplish this, the PMGRC enrolls probands (individuals with a suspected genetic disorder) who do not have a clear diagnosis from standard-of-care molecular testing, along with those probands’ biological parents and/or siblings.

Though chatbots have been implemented successfully in a variety of clinical settings, to our knowledge this is the first analysis of using chatbot technology to facilitate complex informed consent for enrollment in a genomics research study.^7–9^ Here, we report results from a case study where we examine and reflect upon this experience in the utilization of a chatbot for a large-scale genomics study. We lay the foundation for future clinical studies to demonstrate noninferiority or superiority.

## Materials and methods

### Chat platform

**Figure 1.**
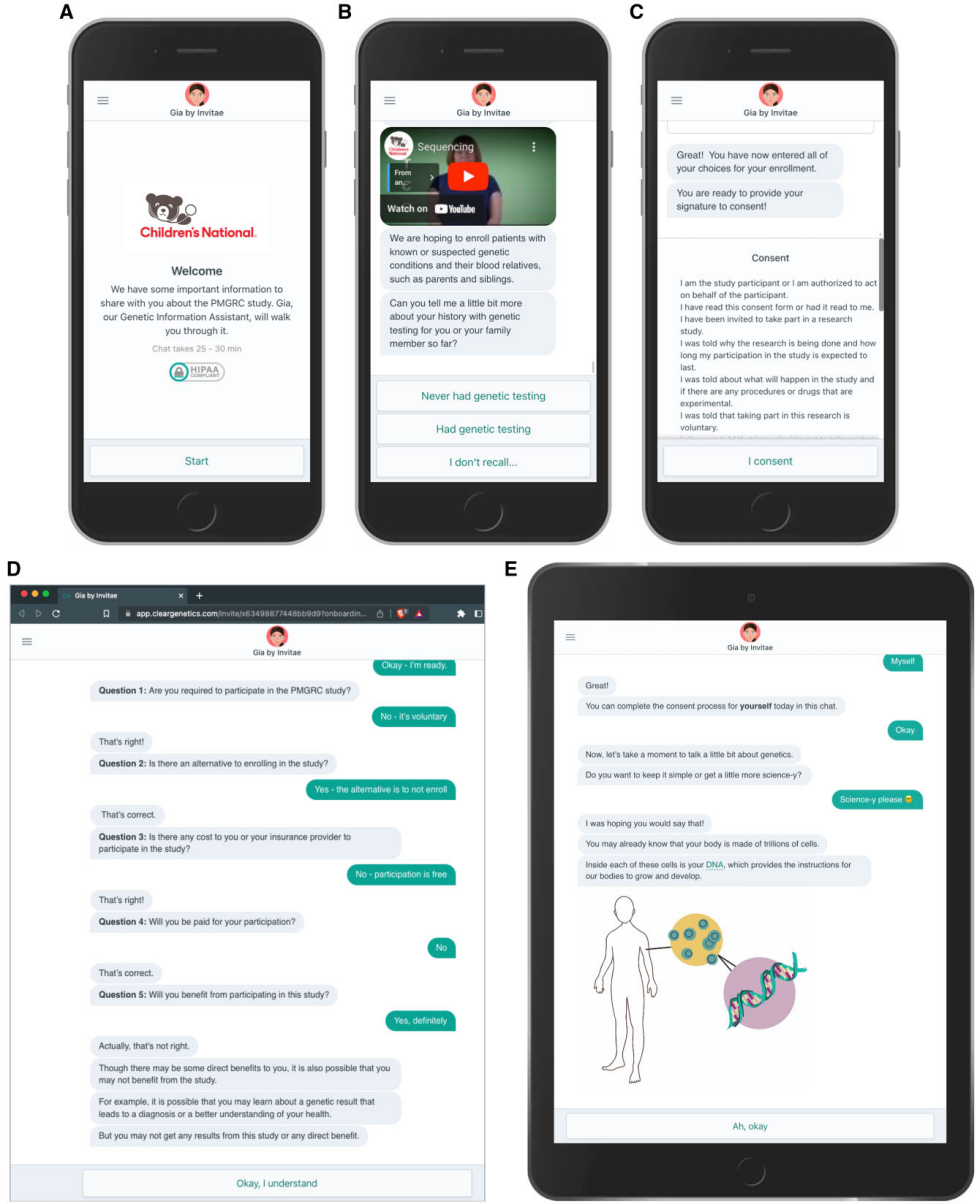
Examples of Gia chat interactions and accessibility. (A–C) Chat using a mobile device and highlighting HIPAA compliance, educational video link outs, and the final consent. (D) Chat using a desktop web browser and the built-in teach back for incorrect quiz answers. (E) Chat using a handheld tablet showing the ability to select more in-depth information about genetics. Link to interface demonstration: https://app.cleargenetics.com/invite/x63498877448bb9d9?onboarding&demo&noresume.

Chat-based consent was developed using the HIPAA-compliant Genetic Information Assistant (Gia^Ⓡ^). Gia chats presented scripted content that allowed users to interact with the chat through prepopulated responses (Figure 1). Since the chats were web-based, private health data was not stored on the user’s device. Each user’s conversation progress was saved in their unique chat encounter, so individuals could complete the consent at their convenience. The interface presented prescripted text conversation (Supplementary File S1) and is designed to be engaging, empathetic, and upbeat, qualities that are shown to increase user engagement and communication efficacy.^10^

### Institutional Review Board (IRB) approval of consent chat script design

The script for the chat-based consent was developed by the study genetic counselors and geneticists based on the content of the existing IRB-approved research consent (Protocol Pro00015852). The chat was designed to offer a flexible user experience, with essential content presented to all users and branching logic that presents optional content only when specific information was requested.^11^ This outline was then used to develop a detailed, conversational script. The script was assessed to have an eighth-grade readability level (Flesch-Kincaid Grade Level = 8, Flesch Reading Ease = 62.1). It was reviewed and approved for use by the CNH IRB and can be viewed as Supplementary File S1.

### GREGoR site activity, participant inclusion criteria, and entry into the study

**Figure 2.**
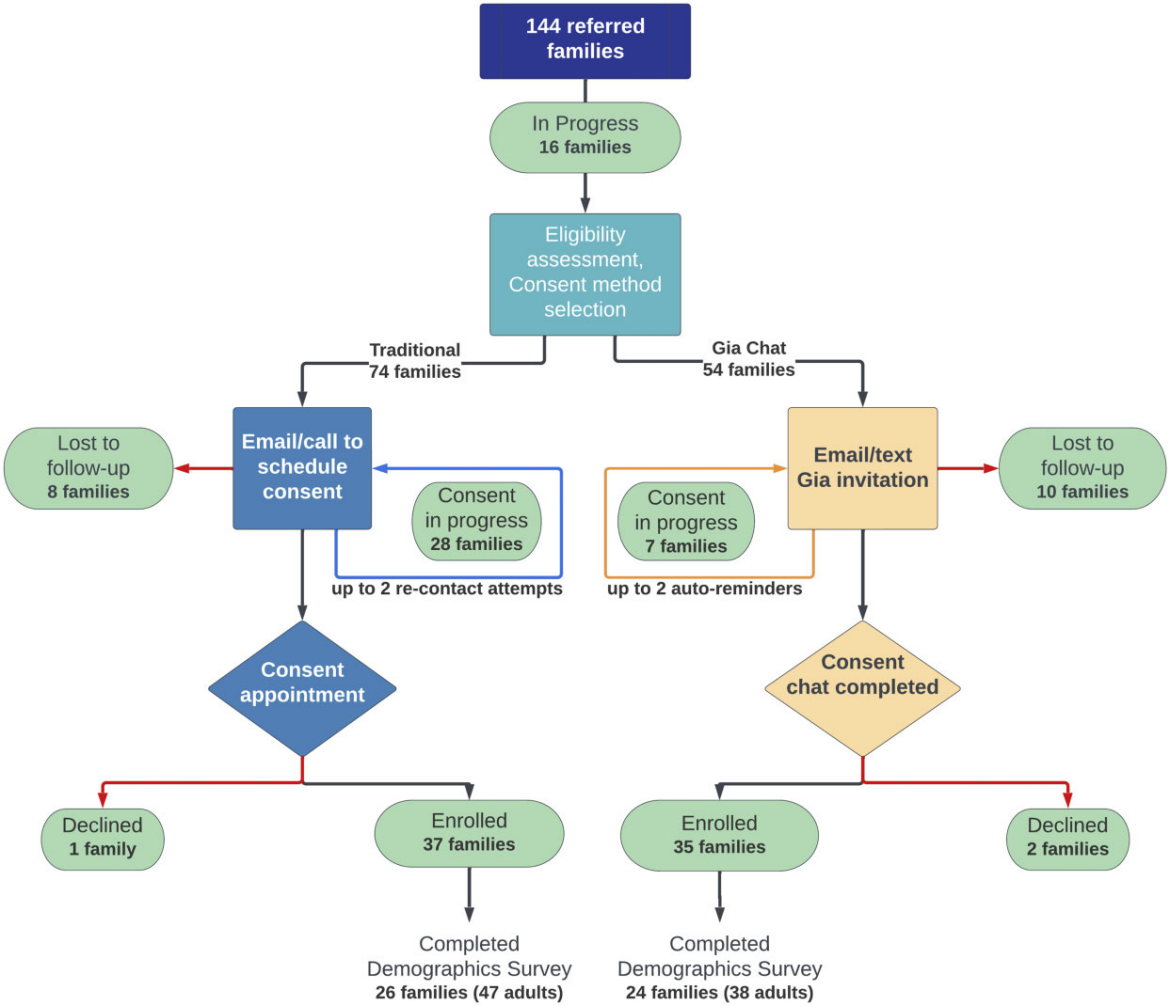
Consent status for referred families at the time of data collection. A flowchart showing the progression from referral to completion of consent and the demographics survey. Ovals indicate the current status of referred families. This analysis includes the first 6 months during which the chat-based consent was offered, June-December 2022.

We studied the outcomes of 2 informed consent processes over a period of 6 months, from the launch of the chat-based consent option in June 2022 until the end of December 2022. At the time of referral to our GREGoR site, each family was given the chance to choose traditional consent with a study team member or chat-based consent as their entrypoint to participation (Figure 2). Participants were included in this study if they, or their immediate family members, had an undiagnosed suspected Mendelian condition. All other persons were excluded.

At the point of REDCap referral, prospective participants were given a choice: to schedule a time to speak with someone about the project in an informed consent conversation, or to receive a link to go through the consent with a chat-based tool online (Figure 1).

### Traditional consent process

Prospective participants who elected to enter the traditional consent process met with study team members either in clinic or over video call. We made up to 3 attempts to schedule that conversation. During the consent discussion, a team member and prospective participant reviewed the research consent form without a set script. For trios or larger families, multiple family members received the option to have a group consent conversation. Assent for prospective participants 7–17 years of age was sought during the consent conversation. Consent was obtained based on the determination of a trained study team member that could assert the participant was appropriately informed.

### Chat-based consent process and user experience

In the chat-based consent pathway, users were sent a unique link by email and/or SMS. The system would automatically send up to 2 reminders to engage with the chat in weeks 2 and 4. The chat provided users with a brief orientation on how to use the interface and offered the alternative of consenting with a study team member. As users proceeded through the chat, the system notified our team via email of any necessary support or follow-up, including users’ requests to speak directly with a team member.

To ensure participants understood the benefits and risks and did not rapidly click through the consent, we implemented a 10-question quiz to replace the human determination that happened during the traditional consent process. This allowed us to demonstrate to our IRB that the participants were appropriately informed (Table 2). All questions had to be answered correctly for a passing score, at which point consent was finalized. Incorrect answers yielded a response where the error is noted, and the correct answer is explained in detail. At the end of the quiz, the incorrectly answered questions were posed again. Users were given 2 attempts to pass each question, after which the platform sent an email notifying our team to communicate directly with the user.

**Table 2.** Quiz content and responses.

Question	Correct answer	Frequency of incorrect response (on first attempt)
1: Are you required to participate in the PMGRC study?	No—it is voluntary	2/59
2: Is there an alternative to enrolling in the study?	Yes—the alternative is to not enroll	2/59
3: Is there any cost to you or your insurance provider to participate in the study?	No—participation is free	1/59
4: Will you be paid for your participation?	No	0/59
5: Will you benefit from participating in this study?	Maybe, but not necessarily	7/59
6: Are there any risks from participating?	Yes, there may be risks	4/59
7: What is the purpose of the study?	To discover the causes of genetic health issues and better understand the role of genetic variants	1/59
8: Will you need to provide samples and/or data?	Yes, the study team may collect some samples and/or data.	0/59
9: Will your personal identifiable data be kept confidential?	Yes, the study team will work to keep my information confidential	1/59
10: If you are injured as part of this study participation, what can you do?	I can contact the principal investigator	1/59

After using the chat-based consent process, users were presented a multiple-choice quiz, which allowed for 2 attempts to achieve a passing score.

Users were allowed to provide consent for themselves and/or for up to 3 dependent children in a single chat encounter. Each user had to complete their own consent. For prospective participants who were 7–17 years of age, legal guardians could provide their consent, at which point the chat-based consent platform sent an email to our team to set an assent appointment with the proband and legal guardian. Assent was scheduled in the mode of traditional consent.

Finally, we gathered user feedback about the chat consent experience. The final display of every completed consent conversation was the prompt “Rate your experience—We'd love to get feedback on Gia.” Responses were gathered using a Likert scale with 3 faces: happy, neutral, and sad.^12^ This was the only question employed to survey experience.

### Data collection and statistical methods

Data was analyzed for individuals who completed consent. As consent conversations occurred with parents, “family” is the unit analyzed. Chat-based consent users are called out as “users,” since only 1 person could interact with a chatbot, but could consent for their dependent children, and/or activate the assent process for prospective participants.

Traditional consent conversation duration was assessed through Zoom call timestamps for 28 consented families (9 families consented in person) and consent status was collected from REDCap. Data about date and time of chat interactions, responses, and chat status were exported from the Gia platform through a csv download. Data from selected consent type, referral date, consent status, enrollment date, and demographic information for all enrollees were collected and managed using REDCap electronic data capture tools hosted at CNH.^13^^,^^14^

REDCap is a HIPAA-compliant, web-based software platform designed to support data capture for research studies. Statistics were calculated in Python v3.11.1 with packages scipy.stats v1.9.3, numpy v1.23.0, and pandas v1.5.2 from study-generated dataframes. We ran 2-tailed *t*-tests and chi-squared tests with these packages and assessed significance with *P* < .05.

## Results

### Chat-based consent allowed users more flexibility in their consent process compared to traditional consent

There were 74 families who began the traditional consent process and 54 who chose the chat-based consent process (Figure 2).

**Figure 3.**
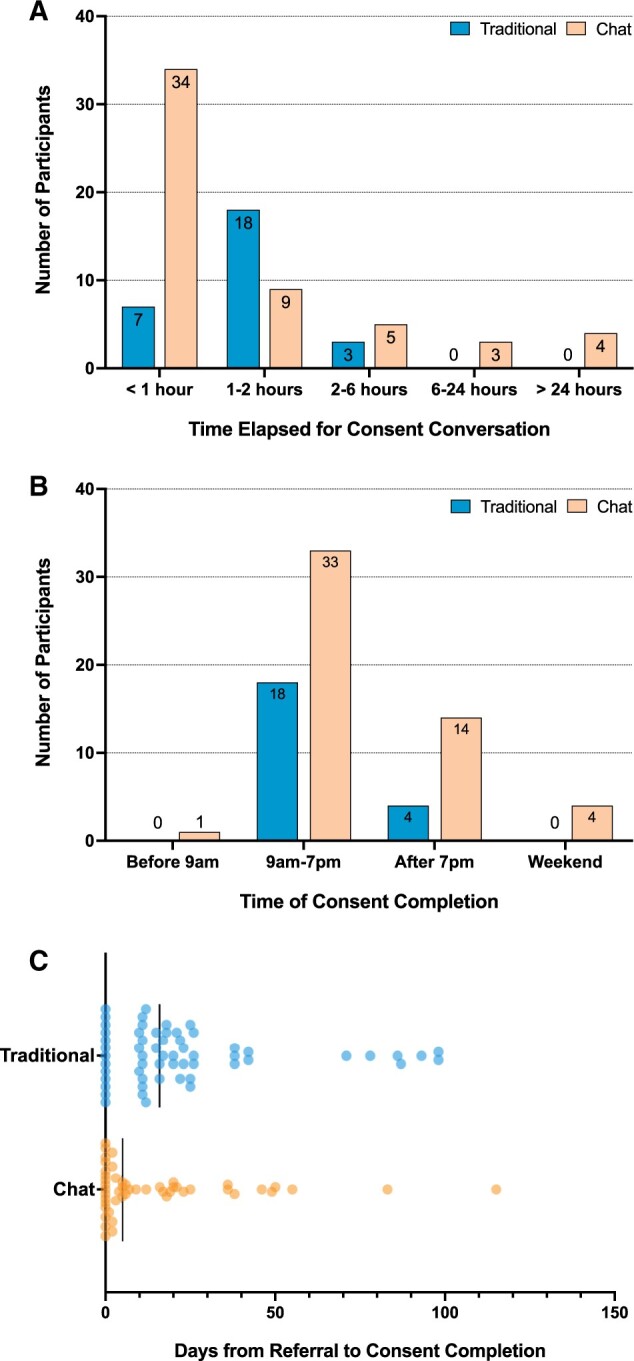
Consent chat engagement patterns. (A) Duration of consent conversation: Amount of time elapsed between opening chat and completing the consent conversation (with an outcome of enrolled, declined, or lost to follow-up). The chat time elapsed to completion includes breaks. For the Traditional method, time displayed is the duration of the uninterrupted video chat. (B) Timing of consent signatures: Proportion of enrollees who signed the consent form before 9 am, between 9 am and 7 pm, after 7 pm, and on a Saturday or a Sunday. Does not include participants who declined or were lost to follow-up. (C) Distribution of the number of days elapsed between referral and consent completion for each enrollee. Using the chat-based consent allowed 16 individuals to enroll on the same day that they heard about the study. Traditional consent was used to enroll 13 individuals on the same day they learned of the study.

Over one-third (37%) of chat-based consent users completed the process outside of 9:00 am to 7:00 pm on weekdays (Figure 3A). Notably, 27% of users completed the conversation after 7:00 pm and 8% on weekends, when study staff are not available.

The median age of enrollees was not significantly different between chat and traditional consent groups (*P* = .84 by 2-tailed *t*-test). There was no significant effect of self-reported sex on consent method choice χ^2^ (1, *n* = 83) = 0.52, *P* = .47. Census race categories of the 2 groups did not differ, with the majority of enrollees in both groups self-identifying as “white” (Table 1).

**Table 1.** Self-reported demographics of enrollees.

Summary of enrollee response	Traditional consent	Chat-based consent
Age (years), median	45	42.5
Age (years), range	18–69	27–72
Self-reported sex, *n* (% of method)		
Female	25 (53)	22 (61)
Male	22 (47)	14 (39)
Race/Ethnicity, n (% of method)		
American Indian/Alaska Native	2 (4.3)	2 (5.3)
Asian	0	2 (5.3)
Native Hawaiian or other Pacific Islander	0	0
Black or African American	2 (4.3)	1 (2.6)
White	42 (89.4)	32 (84.2)
Middle Eastern or Northern African	0	1 (2.6)
More than 1 race	2 (4.3)	3 (7.9)
Unknown	0	0
Hispanic	5 (10.6)	6 (15.8)

Following consent to enroll in the PMGRC study, enrollees were sent a demographic survey. These responses were summarized for responses before December 2022.

### Conversation time was shorter with use of chat-based consent

Traditional consent conversations had a median time of 76 minutes (range 43 - 134 minutes). Most consent conversations were individual video calls (*n* = 18, 64%). In 6 cases, multiple family members joined the same consent conversation: 1 had 5, 3 had 3, and 6 had 3, with a median of 77 and a range of 57–134 min.

The majority of chat-based consent users (62%, *n* = 34) completed their conversation in <1 h (Figure 3B). The median time was 44 minutes. This was significantly less than the median traditional consent time of 76 min (*P* = .0083, 2-tailed Mann-Whitney test). Many chat-based consent users paused the conversation and returned to complete it later, and these breaks are included in the total completion time. Four users (7%) spread the consent conversation over multiple days.

### Time to consent completion was faster with chat-based consent

For the chat-based consent, 69% of families completed the process (35 enrollments and 2 declines) and 51% of families completed the traditional consent process (37 enrollments and 1 decline). There was no statistical difference in the number of families choosing to enroll relative to prospective families in both processes, χ^2^ (1, *n* = 128) = 2.78, *P* = .095 (Figure 2). The remaining families were still in process in December 2022 (35, 7 of whom used the chat) or lost to follow-up (18, 10 of whom used the chat).

The chat-based consent process was associated with significantly faster progression from referral to consent completion (Figure 3C). The median time from referral to consent was faster by 11 days with the chat-based consent (5 days for chat-based consent vs 16 days for traditional consent, *P* = .0222 by 2-tailed Mann-Whitney test).

### High quiz pass rate and no negative experiences reported with chat-based consent

Of the 59 chat users who took the quiz, ∼96% passed. Most (76%) users passed the quiz on the first attempt, and 20% passed on the second attempt. The quiz accurately identified situations in which the user was not able to provide informed consent. Two users failed the quiz twice and, in both cases, a team member followed up with the user or legal guardian. The results of the quiz can be found in Table 2.

For user experience, out of 42 responses, 36 (86%) were positive and 6 (14%) were neutral. This suggests that most users had a positive experience using the chat.

## Discussion

Given limited study staff time, automation tools have great appeal. With the rise of generative chatbots, it is inevitable that artificial intelligence chatbots will be implemented in diverse fields, including medicine.^10^^,^^15^ However, at this time, it is necessary to build chatbots with appropriate guardrails to ensure that the required information is covered and to prevent “hallucinations” or incorrect information presented to a prospective participant. A predetermined, set script enables us to gain the benefits of chatbot consent for our study team while ensuring that participants are appropriately consented.

Our study shows time savings through the use of chat-based consent, both in terms of study staff effort, but also the time from prospective participant referral to completion of informed consent. A novel development of this study was demonstrating the value of manually converting a complex consent document into an IRB-approved chatbot-friendly script. Similar to the Personal Genome Project,^16^ we employed a quiz to assess the understanding of chat-based consent users, which suggests that the decreased time did not prevent knowledge transmission.

The lower percentage of families who completed the traditional consent process suggests that the chat-based consent process is more effective at moving users from the referral point to completion, either enrollment or declination. The chat-based consent also allowed for same-day consent. For example, study staff shared the chat-based consent links at a family advocacy conference, which enabled a single staff member to enroll patients through a chatbot while they attended the conference, rather than missing sessions for enrollment through the traditional consent process. While this result is encouraging, there could be other factors that contribute to this efficiency, such as the participants willingness to engage at a conference, and more careful study between the different consenting methods is warranted.

Study staff also have clinical duties and are not uniformly available throughout the week. The chat-based consent tool alleviated the burden on their clinic schedules. Anecdotally, staff used their increased availability to respond to participant questions, investigate prior testing history and symptoms to determine study eligibility, and to participate in GREGoR consortium-wide conference calls and events. This is a positive development for the quality of participant enrollment (more time spent determining eligibility) and for attendance in these important technical, GREGoR consortium-wide meetings.

### Limitations

The majority of the enrollees who responded to our demographic survey were self-identified white. Future improvements to the chat-based consent method will include additional languages, to better represent the communities served by PMGRC.^17^^,^^18^ We could only assess the experience of adults capable of providing consent, and future studies will be needed to expand chatbot access to children ages 7–17 for the provision of assent. Future studies should include the experience of those who participate via assent.

Even though most users passed the chat-based consent quiz with 2 attempts, we have no data to which this pass rate can be compared, since quizzes or other structured knowledge checks are not currently part of the traditional consent standard of practice. However, the success of the quiz points to a more rigorous future where quizzes might be incorporated in consent forms. This would help to ensure that informed consent is indeed informed.

While neither a noninferiority or superiority trial, there is still a critical need to consider the place of chatbots in a postgenerative chatbot world and our case study identifies some considerations for such trials. Critically, in-person conversations will need to be timed so that they can be compared to online, timestamped consent conversations and in-person conversations should also be assessed via the same quiz. Trials of that nature will allow for a valid assessment of the superiority or noninferiority of a chatbot.

## Conclusion

Chatbots are becoming commonplace. Realizing their benefits can be tempting in the research setting, but there must be significant ethical considerations. We circumvented some of these through use of a scripted, rather than generative, chatbot to alleviate a known bottleneck in research. This had the 2-fold benefit of flexibility for prospective participants to have a consent conversation on their own time, while ensuring IRB oversight of the entire process through the script.

We have included our script in the Supplementary Material to help other teams design their own chatbots to realize the benefits of the time-saving aspect of chatbot technology. Future studies can be designed in light of our foundational experience as they work to assess the superiority or noninferiority of chat-based consent. We hope that these studies will have an emphasis on quizzing participants in traditional and chat-based consent groups. With an answer in hand, the fields will be positioned to consider the place of generative chatbots in the informed consent setting, potentially opening the door to teach-back from human to generative chatbot to assess understanding.

While ethical, legal, and social implications of research scholars consider generative tools and whether a chatbot going “off script” is more concerning than an imperfect human interaction, scripted chatbots provide the lion’s share of benefits to research participants and teams, while assuaging real concerns of IRBs.

## Supplementary Material

ocad181_Supplementary_Data

## Data Availability

The data underlying this case study cannot be shared publicly to protect the privacy of individuals who participated in the study. Data will be shared on reasonable request to the corresponding author.
