# Supplementary material for: Using a chat-based informed consent tool in large-scale genomic research
Source: J Am Med Inform Assoc. 2023 Sep 4;31(2):472–8. doi: 10.1093/jamia/ocad181 (PMC10797258; doi:10.1093/jamia/ocad181)
Supplement: ocad181_Supplementary_Data [file ocad181_supplementary_data.docx]

Table of contents:

- [Greeting](#kix.gg6rs6chvvz)
- [Consent document](#r6dno1mlo4ee) - **consent doc**
- [Option to continue with Gia](#cvikcajdhw07)
- [Who is considering enrollment?](#cmg3q8eue1tu)
- [Genetic background](#kix.lngr1rki7rin)
- [Genetic variants intro](#9qgmj9w0tgit)
- [Enrollee’s experience with genetic testing](#mymdxq1s4r9u)
- [Goals of the study](#fs8xawo87w9)
- [Voluntary](#kix.lpmpa1ak0mg5)
- [Free!](#a8qdnl31ng2)
- [Background - who is involved?](#t3gy30h22bw5)
- [Who is being recruited/enrolled?](#f6i4t51digoe)
- [Research testing doesn’t replace clinical testing](#l5cc5ivdzwxl)
- [What happens once enrolled?](#j0yeitinrvzx)
- [Study information collected](#y5y0o1tuihx0)
- [Data access/use/privacy](#4fwij2vcp5pm) - **Notice of** **Privacy Practices**
- [HIPAA](#kix.4szgl156wckc) - **HIPAA doc**
- [Certificate of Confidentiality](#2oeym9cx7hca)
- [Result types](#gb1ho3hjv32n) - primary and secondary, medically actionable vs. not
- [Incidental results](#h9vzaynehorz)
- [Duration of study](#9q9rolbmevgg)
- [Revoke or withdraw](#5irj7sxjzv8e)
- [GINA](#jna1e18w795s)
- [Risks](#iqdz2npcfyar)
- [Benefits](#212w0t37u2rd)
- [Contact for questions or concerns](#kix.md0r64g4ncj3)
- [Ready to decide about consent?](#kix.4wyhitpdo9u6)
- [Quiz](#h0o5unw7v99c)
- [Consent options](#ddf6sulrvwni)
- [Signature for “self”](#i68rsx15waw0)
- [Signature for “child”](#kix.pie2e8onu7j3)
- [Next steps](#6k9n9g7y8c8r)

| Gia: {timeOfDayGreeting()}, {patientFirstName}!  Gia: I’m Gia, your automated Genetic Information Assistant from the **Pediatric Mendelian Genomics Research Center (PMGRC)** through Children’s National, UC Irvine, and Invitae.  Gia: I’m here to tell you all about the PMGRC study and help you decide if you want to participate.  Patient: Hi Gia! 👋  Gia: I’m going to provide details about the study like why we are doing this research, what you will be expected to do if you enroll, and possible risks and benefits.  Gia: My job is to give you plenty of information to help you choose whether or not to participate.  Patient: Great! | | |
| --- | --- | --- |
| [if invited via individual invite link] | [if invited via Invite Generator link]  Gia: To start, please provide your basic info so we can contact you if needed.  Patient: [Form: First, Last, DOB, Email and/or Phone]  Gia: Thank you, {name of person}! | |
| Gia: This chat usually takes about 30 minutes to go through before you can sign the consent form to enroll at the end.  Gia: You can always pause and come back at a later point.  Gia: At any time during our conversation, you can click the menu button (☰) at the top left to find chat options.  Gia: If you want to change any of your responses, just click on the response or go to the menu.  Patient: Okay / I understand  Gia: All of the information I will share is also described in more detail in our consent document.  Gia: You can only sign the consent form electronically after reviewing it with me or with a study team member.  Gia: You can access an example of the consent document by clicking this link, but please remember to return here to complete our chat.  [link to PDF of Consent: <https://documentcloud.adobe.com/link/review?uri=urn:aaid:scds:US:c2c31666-e549-4415-8bf1-c0d1b822242b>]  Patient: Can I look at this after we chat?  Gia: Yes, you can come back here to access the link later, and I can also provide the link again at the end of the chat.  Patient: Okay, great  Gia: Whether or not you choose to participate in the study, the team at Children's National may review your interactions with me, Gia, to better understand how people interact with me and how I can improve.  Patient: Ok, sounds good  Gia: If you are ready, we can start going through the information together right now!  Gia: If you would rather review the consent later or you would rather review it with a member of the study team, just let me know! | | |
| Patient: I’d like to continue chatting with Gia now.  Gia: Okay! | Patient: I want to pick up this chat with Gia sometime later.  Gia: Okay! No problem.  Gia: You can use the link in your text message to come back any time.  Gia: Let me know when you are ready to chat.  Patient: I’m ready to chat now | Patient: I’d prefer to talk to someone from the research team.  Gia: Sure thing.  Gia: Someone from our study team will contact you directly by phone or email within the next 2-3 weeks.  Gia: Please provide your preferred contact information.  Patient: [Phone and email entry field]  Gia: Great, thanks!  Gia: You should hear from the team soon.  Gia: If you have questions now, you can contact us at [PMGRC@childrensnational.org](mailto:PMGRC@Childrensnational.org)  [Go to end of chat/chat complete] |
| Gia: Let’s do this!  Gia: Please be sure to take notes and write down any questions you have as we go through this chat, as you will have the option to talk to a study team member at the end. ✏️  Gia: If you have any questions that are not answered during this chat, we will put you in touch with a team member to discuss them further.  Patient: Sounds good! 👍  Gia:The goal of this study is to help identify the causes of genetic health conditions.  Gia: You are being invited to join this study because you or someone in your family has a genetic health condition (or a health issue that might be related to genes).  Gia: We often enroll multiple family members (people related by blood) in the study. | | |
| Patient: Okay | Patient: Why only those related by blood?  Gia: Having genetic data from blood relatives can help us do a better job of interpreting genetic findings.  Gia: For example, if we find a genetic change in a person who has a health issue, it is important to know if this genetic change was passed down through the family.  Gia: So, having data from blood-related family members helps us interpret what we find.  Patient: Ah, that makes sense. | |
| Gia: Ideally, we would enroll **the person with the genetic health condition** as well as **both of their biological parents** (mom and dad), if possible.  Gia: So, if one or both parents is able to enroll, that would be great!  Patient: Good to know!  Gia: Each person who enrolls in the study will have to go through this study consent process.   Gia: You can enroll yourself and/or your children in this chat today, and we can send a separate chat link to any other adults who want to enroll.  Patient: Okay  Gia: For children who are 6 years old or younger, a parent or guardian can complete the consent on their behalf.  Gia: For children ages 7 to 17, (or for adults who are under another person’s legal guardianship), a parent or guardian must complete the official consent.  Gia: We will also need to talk directly to the child (if they are capable) to get their agreement about participating.  Gia: People who are age 18 or older need to go through the consent process themselves (except those who are under another person’s legal guardianship).  Patient: Okay / I understand  Gia: Can you tell me who in your family might consider enrolling in this study? (check all that apply, including any parents or other adults who might enroll)   - Myself - My child/children - My child’s other parent - Another adult family member   [if “myself” is selected…]  Gia: Great!  Gia: You can complete the consent process for **yourself** today in this chat.  Patient: Okay  [if “my child’s other parent” or “another adult family member” is selected…]  Gia: If your child’s other parent or another adult family member is considering enrolling, they should also complete this consent chat themselves.  Gia: If they have not already received a link to this consent chat, I can send them one now with just a few pieces of information. | | |
|  |  |  |
|  |  |  |
| Patient: They have already received a link to this chat  Gia: Okay, great. | Patient: I’d like to send them a link to the chat  Gia: Okay!  Gia: Can you tell me their name, date of birth, and a cell phone number and/or email address?  [Form]  Gia: Thank you! We’ll contact them with the information you've provided.  Gia: If you have other adult family members who are considering enrolling, please let our study team know by emailing us at PMGRC@childrensnational.org.  Patient: Okay, thanks![loops back to this section] | |
| [if “my child/children” selected]  Gia: How many of your children are you considering enrolling?  Patient: One child / More than one child  Gia: Okay, can you please select the ages of your children?  Patient:   - 6 years or younger - 7-17 years - 18 years or older   [if “6 years or younger” is selected]  Gia: Okay  Gia: **For your child who is under age 7 years**, you will have the option to complete their consent and enrollment during our chat today.  Patient: Great, thanks!  [if “7-17 years” is selected]  Gia: **For your child who is between age 7 and age 17,** you will have the option to give your consent for your child to enroll in the study.  Gia: But because your child is over age 6, we may also need to talk to them briefly to tell them about the study.  Gia: If your child is developmentally not able to communicate, you can let us know when you talk with our study team.  Patient: Okay  Gia: So, after you complete the consent chat with me, I can help arrange a time for you (and your child) to talk to a member of our study team to finalize your child’s enrollment.  Patient: That sounds good, thanks.  [if “18 years or older” is selected]  Gia: Is **your 18+ child** still under your legal guardianship? | | |
|  |  |  |
| Patient: Yes  Gia: Okay  Gia: After our chat today, you will have the option to give your consent for your child to enroll in the study.  Gia: But because your child is over age 18, we may also need to talk to them briefly to tell them about the study.  Patient: Okay  Gia: So, after you complete the consent chat with me, I can help arrange a time for you (and your child) to talk to a member of our study team to finalize your child’s enrollment  Gia: If your child is developmentally not able to communicate, you can let us know when you talk with our study team.  Patient: Okay, thanks. | Patient: No  Gia: Individuals who are 18 years or older need to complete this consent chat themselves.  Gia: Does your 18+ child already have a link to this chat? | |
|  | Patient: Yes  Gia: Okay, then they will be able to use that link to complete the consent chat themselves.  Patient: Okay, thanks for letting me know | Patient: No, can we send them a link?  Gia: Sure!  Gia: Can you tell me your child’s name, date of birth, and a cell phone number and/or email address?  [Form]  Gia: Thank you! We’ll contact them with the information you've provided.  Gia: If you have other adult family members who are considering enrolling, please let our study team know by emailing us at PMGRC@childrensnational.org.  Patient: Great, thanks! |
| Gia: You’re welcome 😀 [common landing after “my child” section, to route to other options correctly]  Gia: Now, let’s take a moment to talk a little bit about genetics.  Gia: Do you want to keep it simple or get a little more science-y? | | |
| Patient: Science-y please 🤓  Gia: I was hoping you would say that!  Gia: You may already know that your body is made of trillions of cells.  Gia: Inside each of these cells is your DNA, which provides the instructions for our bodies to grow and develop.  [Tooltip: DNA stands for deoxyribonucleic acid.]  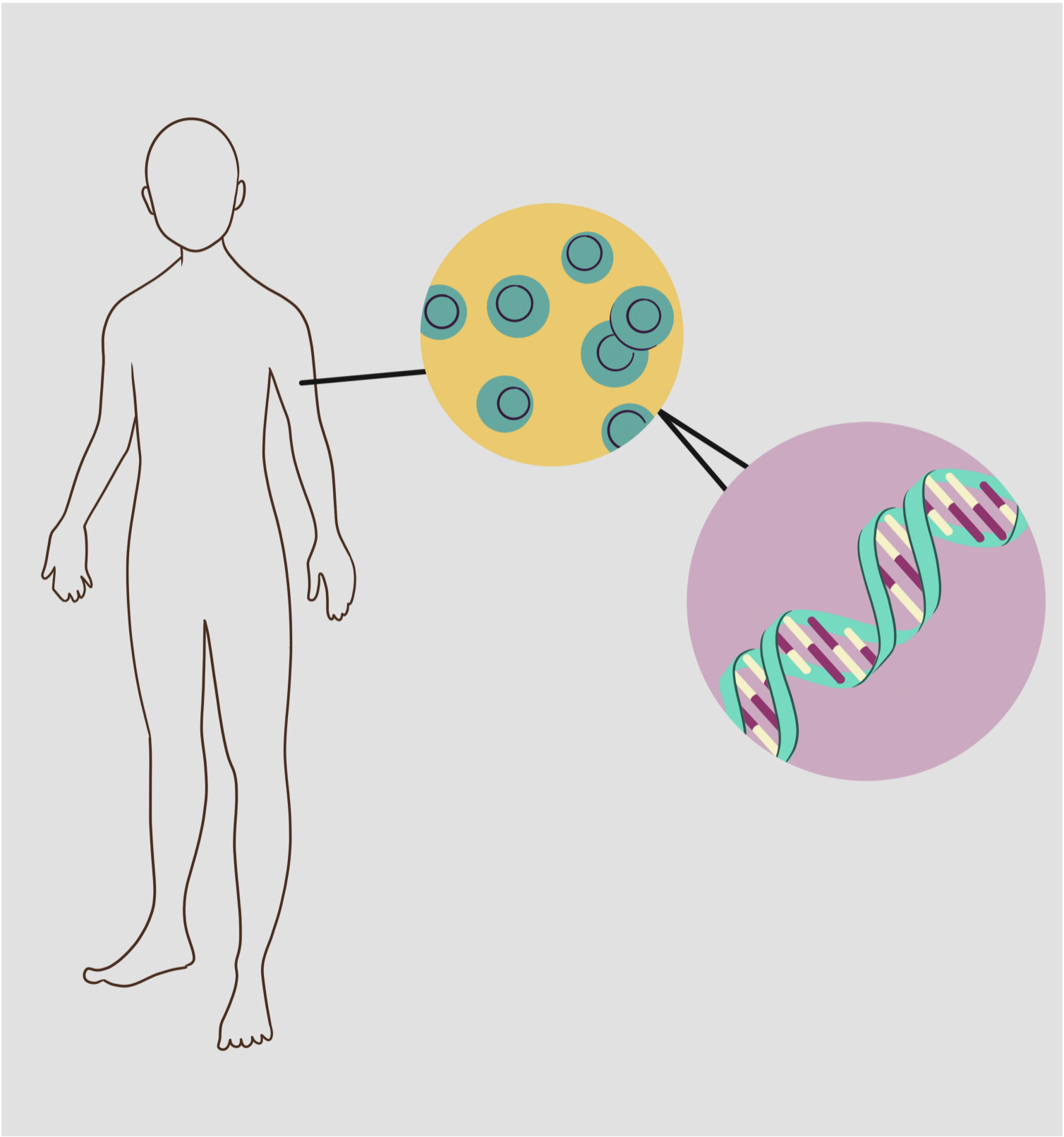  Patient: Ah, okay  Gia: Your DNA is made up of a genetic code, which is represented by four letters: A, T, C and G.  Gia: A little section of the code might look something like this:  ATGCGCTAGCTCCTAGCTAGCCTAGCTAGCTAACGCTAGCCTCGTAGACTACGATCGCTAAGCTAGC  Gia: The code is actually over 3 billion letters long!  Patient: That’s complicated  Gia: I know! But your cells know how to read this code.  Gia: The cells use your genetic information like a recipe book.  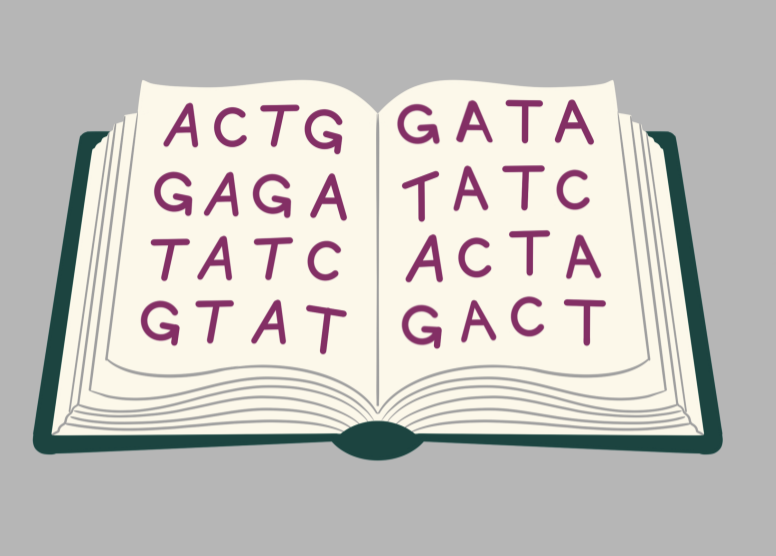  Gia: It is organized into specific sections, called genes (just like a recipe book is organized into individual recipes).  Patient: This sounds familiar / Tell me more  Gia: In a recipe book, each recipe gives instructions for making a different dish.  Gia: In your cells, each gene provides the instructions for molecules called RNA, and each RNA makes a different protein.  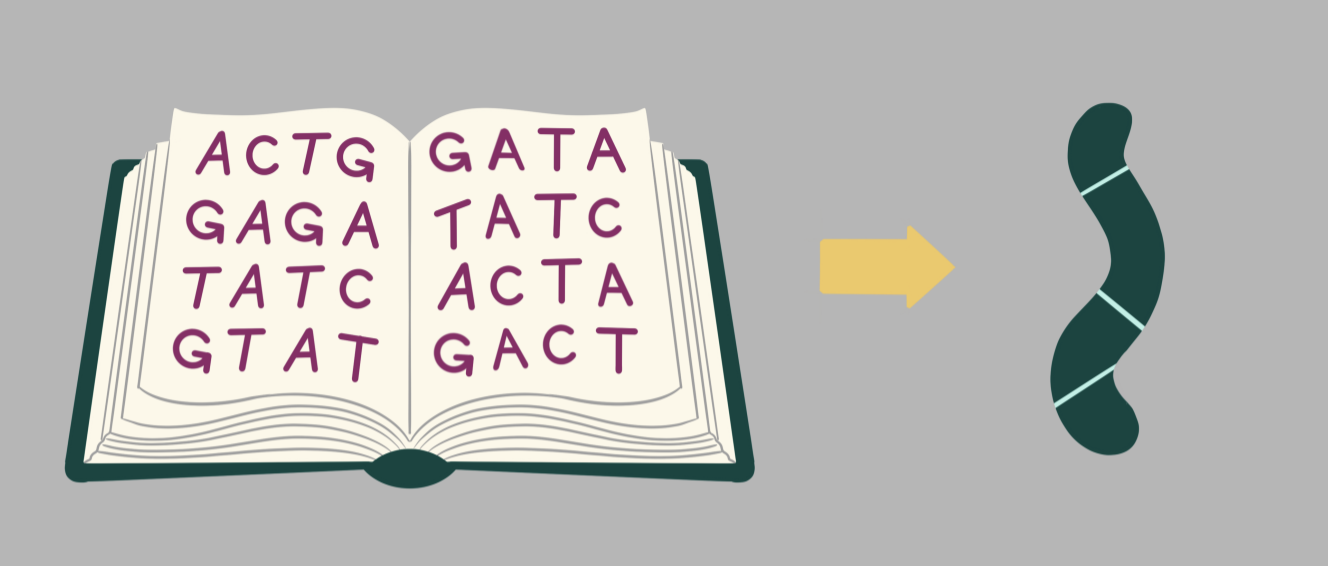  Patient: That’s interesting  Gia: These proteins can help your body to do many important things.  Gia: For example, some proteins build our tissues, like muscles and nerves. Other proteins help our bodies break down food and make energy.  Patient: That's cool!  Gia: I know, right?  Gia: You have 2 copies of each gene - one from each of your parents.  Gia: The 2 copies work together to make you who you are.  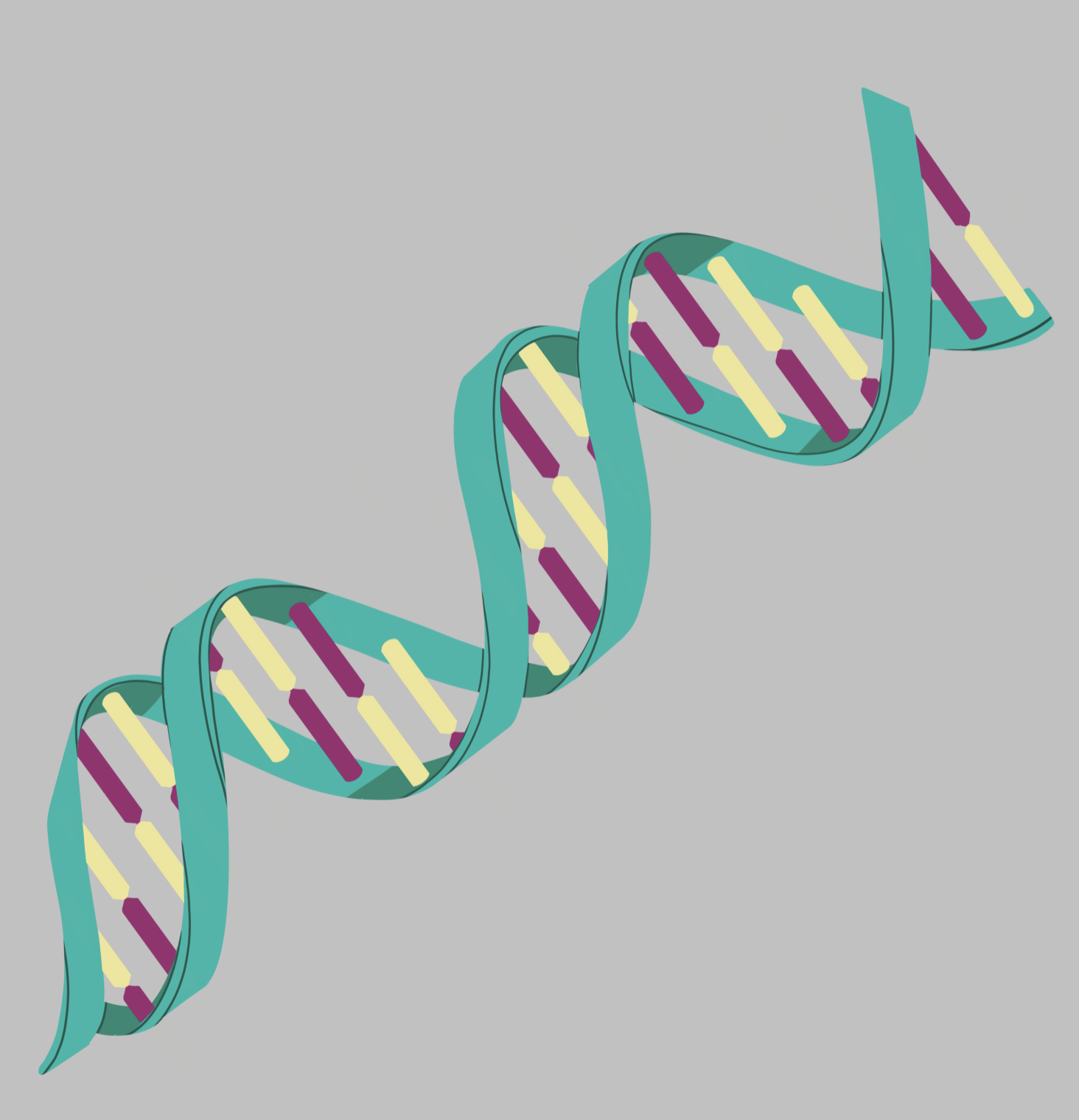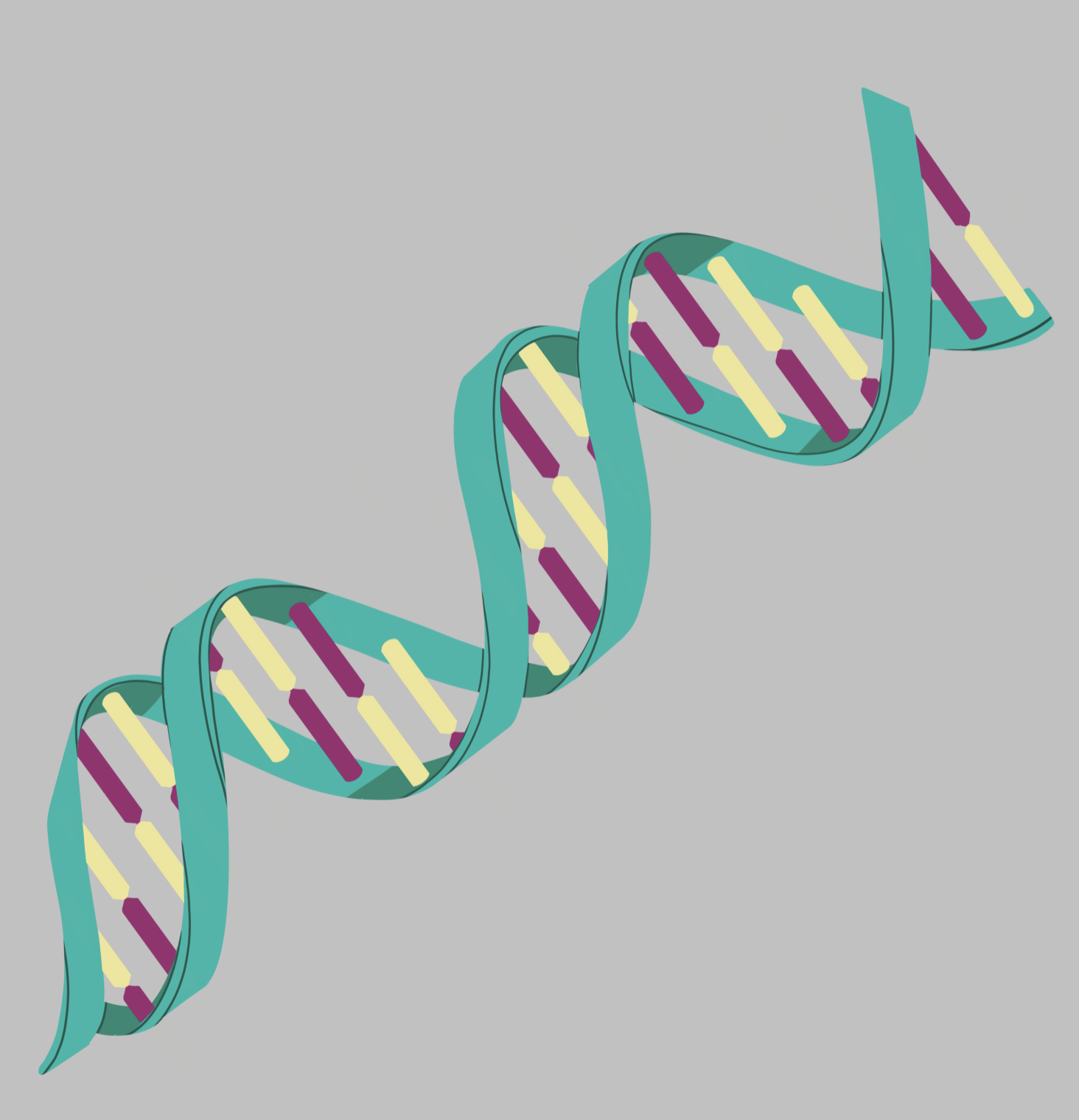  Patient: Got it  Gia: People can have different versions of the same gene. This is what makes each person unique.  Gia: We call these different versions of a gene “variants”.  Patient: What is a variant?  Gia: Remember how we said your genes are like recipes?  Gia: Well, if there are any little typos or slight changes in the recipe, the dish might turn out differently than you expected.  Gia: A variant is when the letters of the genetic code are out of order or changed in some other way.  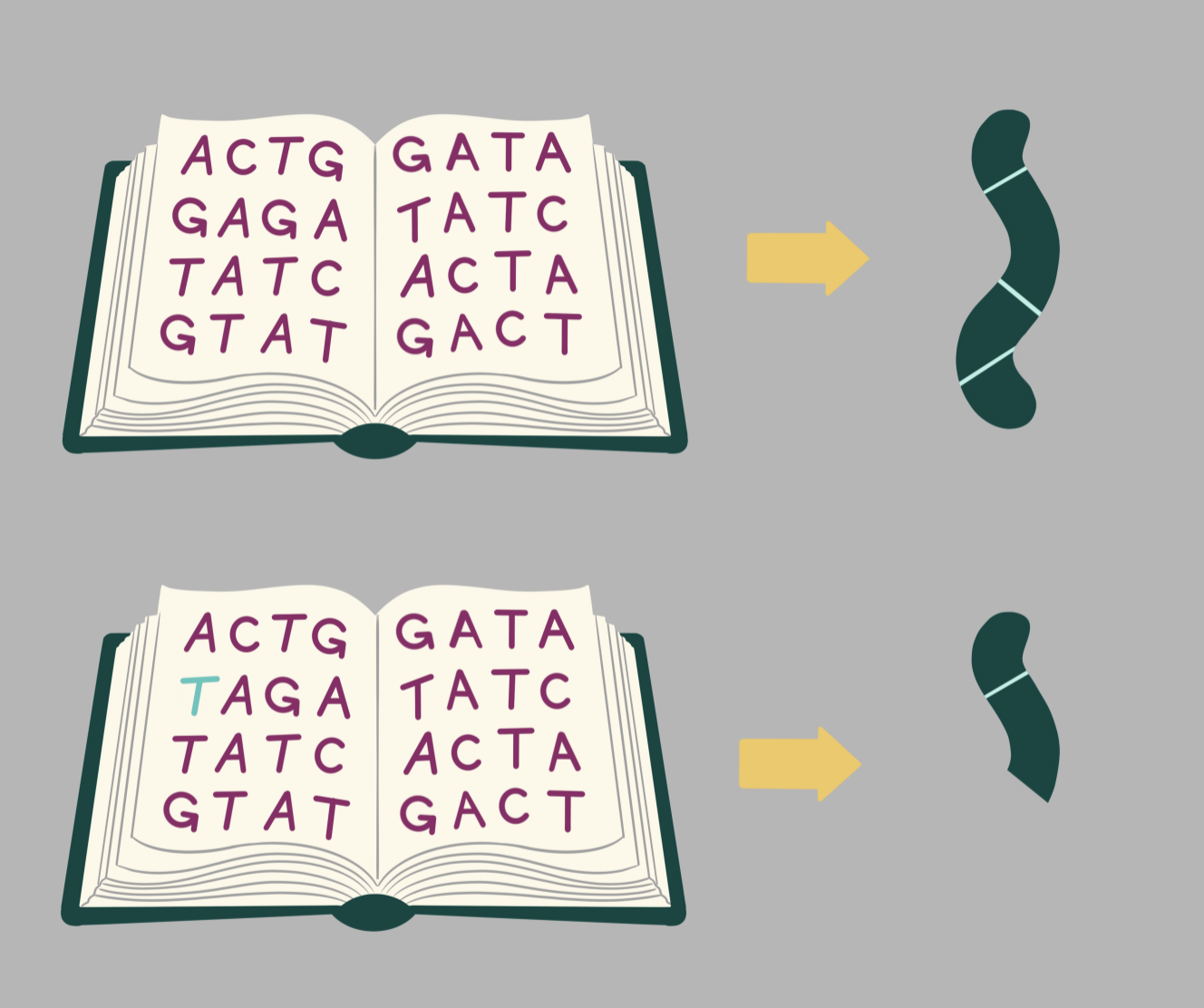  Patient: I see  Gia: When a gene has a variant it gives different instructions.  Gia: Imagine that you are baking a cake and the recipe tells you to use salt instead of sugar, or strawberries instead of chocolate!  Gia: That’s going to affect the cake!  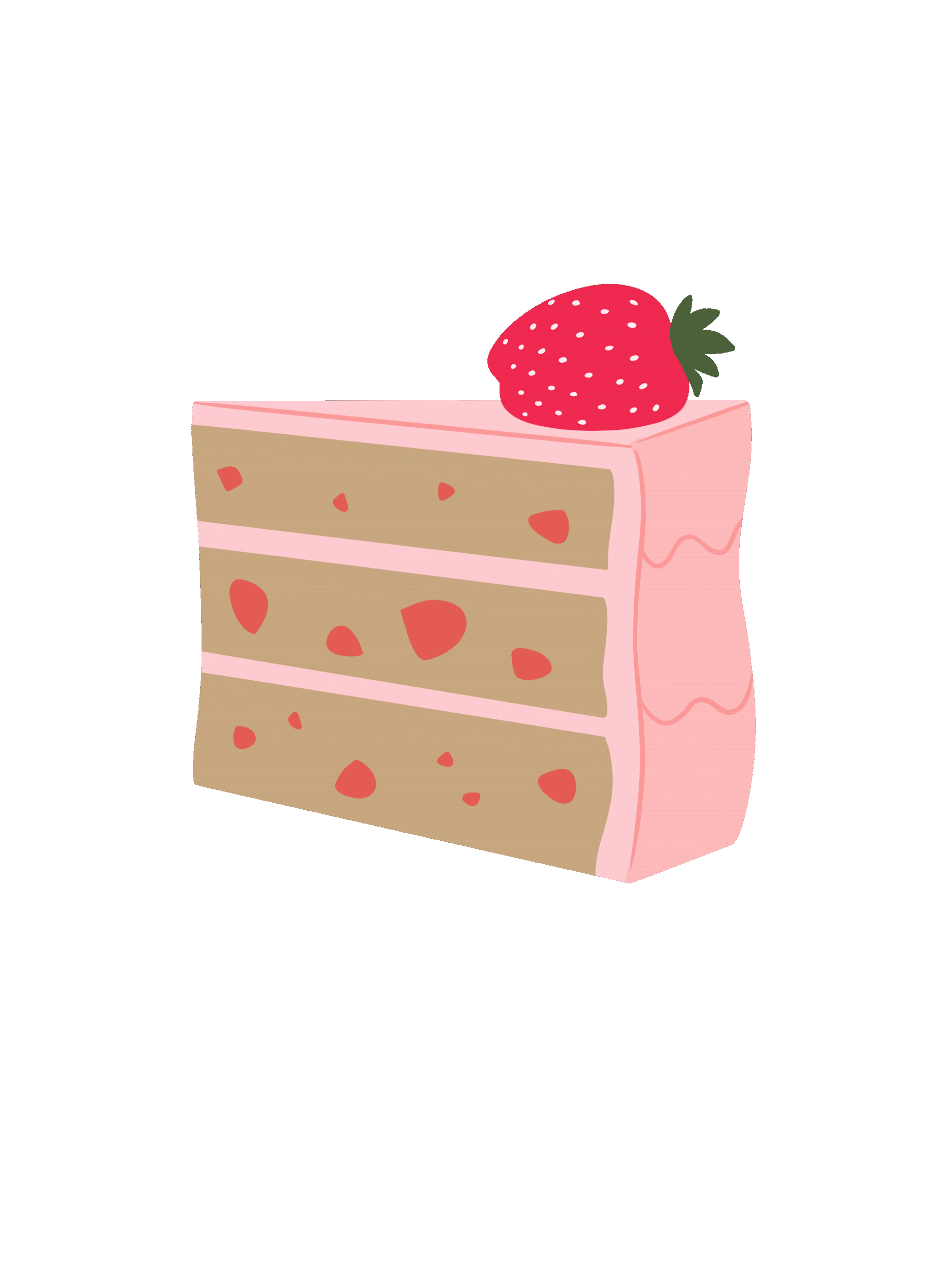  Patient: Got it | Patient: Let’s keep it simple  Gia: Okay, sounds good.  Gia: You may already know that your body is made up of trillions of cells.  Gia: Inside each of these cells is your DNA, which provides the instructions for our bodies to grow and develop.  [Tooltip: DNA stands for deoxyribonucleic acid.]  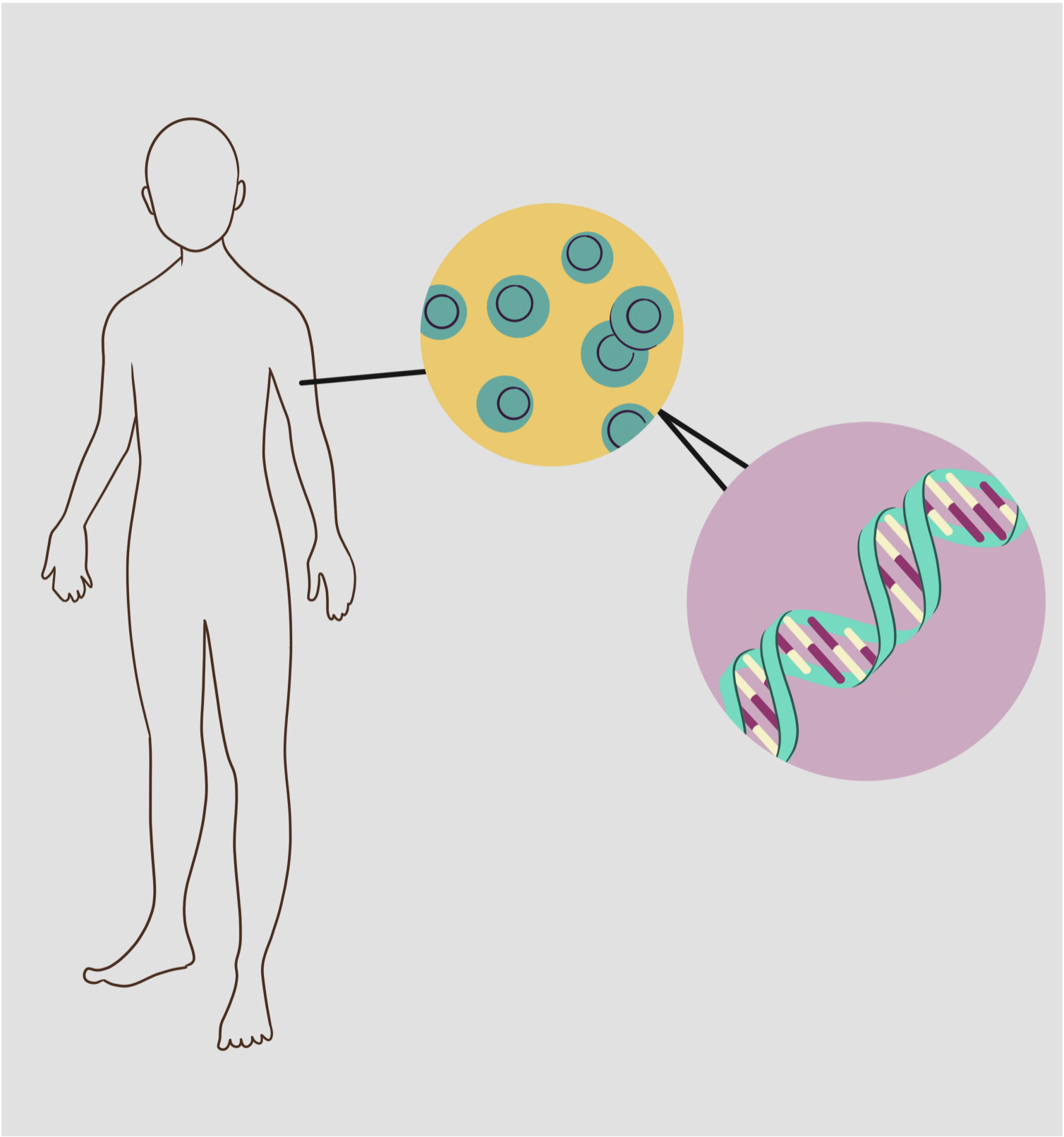  Gia: The DNA is arranged in sections called genes.  Patient: Okay / This sounds familiar  Gia: Genes are a big part of what makes you who you are.  Gia: In your cells, each gene provides the instructions for molecules called RNA, and each RNA makes a different protein.  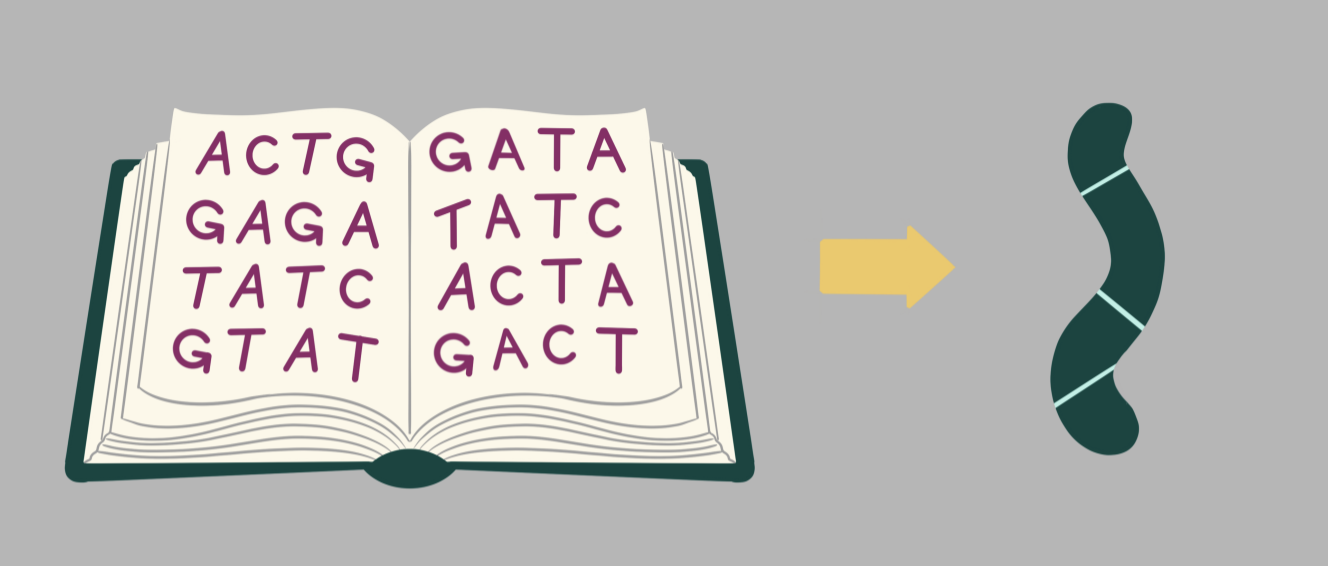  Patient: That’s interesting  Gia: These proteins can help your body to do many important things.  Gia: For example, some proteins build our tissues, like muscles and nerves. Other proteins help our bodies break down food and make energy.  Patient: Okay / That's cool!  Gia: We all have small differences in our genes, which we call “variants”.  Patient: I see  Gia: When a gene has a variant it gives the body different instructions. | |
| Gia: Genetic variants are responsible for making us all unique and different.  Gia: For example, things like eye color are determined by variants in our genes.  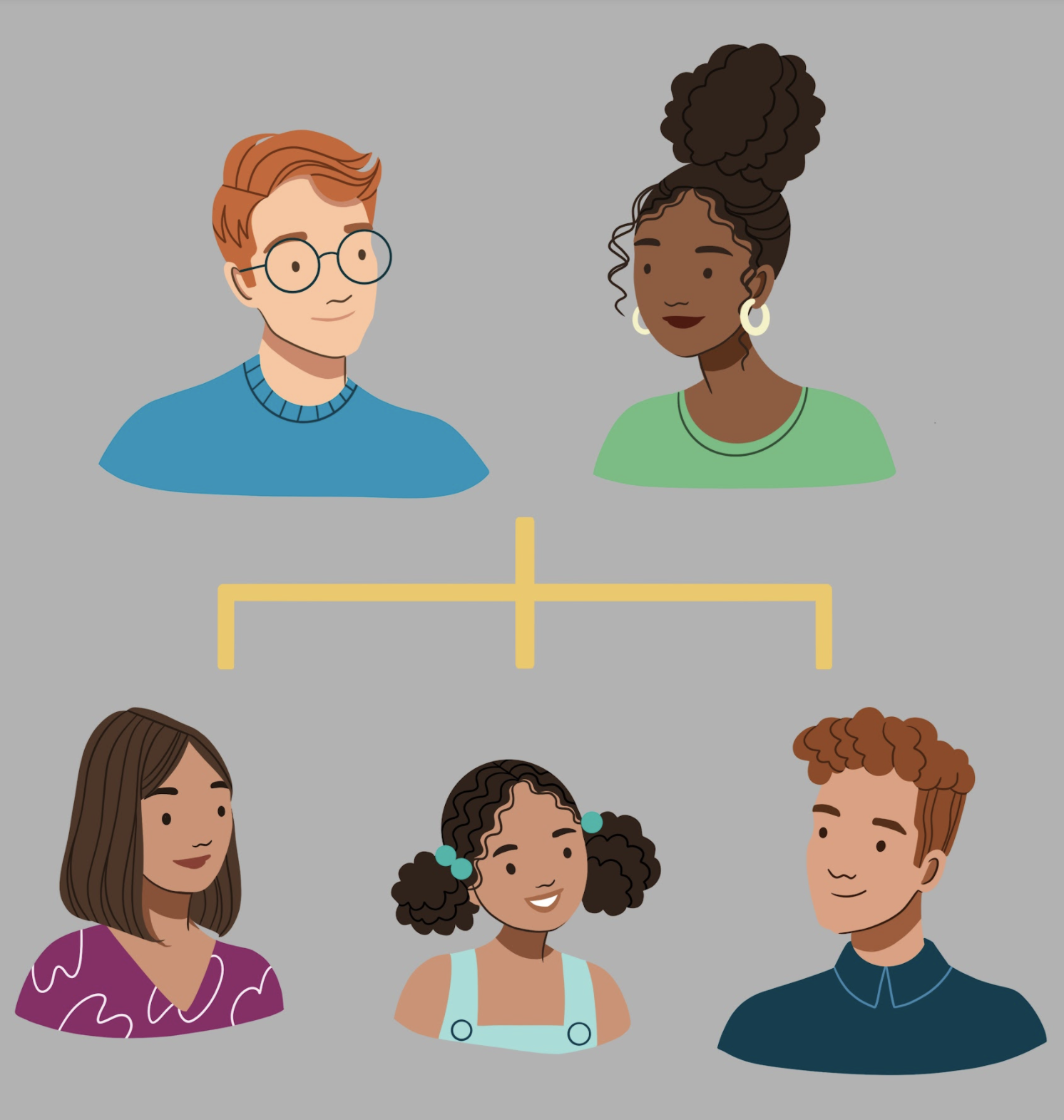  Gia: Sometimes, having a certain genetic variant can lead to health issues.  Gia: For example, if a person has a variant in a gene that is related to kidney health, then their chance of having kidney disease may increase.  Patient: Interesting / That makes sense  Gia: When someone has a health issue caused by a genetic change (or “variant”), we call this a genetic disorder.  Gia: When we suspect that a patient has a genetic disorder, we will often do genetic testing to try and find the change (or variant) that is causing the health issue.  Gia: In the PMGRC study, we are interested in doing different kinds of genetic testing to take a detailed look at people’s genetic material to look for changes that might explain their health issues.  Patient: Great  Gia: Here’s a 1-minute video that talks about one common type of testing, called sequencing.  [embed video  <https://childrensnational.org/news-and-events/video-gallery/sequencing>]  Gia: We are hoping to enroll patients with known or suspected genetic conditions and their blood relatives, such as parents and siblings.  Gia: Can you tell me a little bit more about your history with genetic testing for you or your family member so far? | | |
| Patient: Never had genetic testing / I don’t recall…  Gia: No problem. | Patient: Had genetic testing.  Gia: So you’ve had some experience. That’s good!  Gia: Do you remember what the results were?  Patient: The results found something / The results didn’t find anything / I don’t remember | |
| Gia: One goal for our PMGRC study is to discover **new** causes of health problems, like finding a new gene or new ways that differences in our genetic material can cause health problems.  Gia: If genetic testing didn’t find an answer before, our research *might* be able to find something new.  Patient: Okay...  Gia: Another goal of our study is to better understand prior genetic test results that may have been unclear.  Gia: For example, if previous genetic testing found a “variant of uncertain significance”, our research *might* be able to help explain whether that finding is related to disease or not.  [Tooltip: When a genetic variant is found, but we aren’t sure whether it is linked to a specific health issue, we call this a variant of uncertain significance or VUS.] | | |
|  |  |  |
| Patient: Got it! | Patient: Tell me more about the different types of genetic test results?  Gia: Of course!  Gia: When genetic testing is done, we are usually looking for some genetic variant to explain the patient’s health issues.  Gia: Sometimes, the testing does find a clear answer - a genetic variant that explains the health issue.  Gia: We call this a “positive” result.  Patient: Okay  Gia: If the testing does NOT find any variants that could explain the health issue, we call this a “negative” result.  Gia: Sometimes, the testing can find something that we don’t yet understand well.  Gia: When a variant is found, but we aren’t sure whether it is linked to a specific health issue, we call this a “variant of uncertain significance” or VUS.  Gia: You can also check out the brief video to review the possible types of genetic results:  [embed video: <https://childrensnational.org/news-and-events/video-gallery/possible-test-results>]  Patient: Ah, okay | |
| Gia: We don’t know if this kind of research genetic testing will help people get to a diagnosis, but we’re hoping that it will at least help make science and medicine better.  Gia: This may help other people in the future.  Patient: Well that sounds nice  Gia: We want to make sure you know that joining this PMGRC study is **voluntary**.  Patient: What does that mean?  Gia: You can choose to be part of this study, or choose not to be part of this study. And you can change your mind about being involved at any time.  Gia: Whatever choice you make, the quality of care you receive will not change, and you will not lose access to your medical care or give up any of your legal rights or benefits.  Gia: Also, if we make any changes to the study or if there are any important new findings, we will tell you so that you can decide if you still want to be in the study. | | |
| Patient: Sounds good! | Patient: What if I am a student or employee at Children’s National?  Gia: If you are an employee or a medical or graduate student in training at Children's National, your decision to participate or not participate will not affect your employment or academic standing.  Patient: Great! / How do I let you know if I no longer want to be in the study? | Patient: How do I let you know if I no longer want to be in the study?  Gia: You can contact the study team at any time by emailing PMGRC@childrensnational.org.  Gia: To withdraw from the study, you must send a written letter to the Principal Investigator (Seth Berger) to inform him of your decision.  Patient: Sounds good! / What if I am a student or employee at Children’s National? |
| Gia: Participating in the study is **free** - so there is no cost to you or to your health insurance provider.  Gia: However, if you learn something new about your health as a result of the study, you or your insurance provider will be responsible for the cost of any relevant follow-up testing or medical care.  Patient: Okay  Gia: Just a little more background information for you: Dr. Seth Bergeris the person responsible for this research study at Children's National. He is called the Principal Investigator.  Gia: The National Human Genome Research Institute (part of the National Institutes of Health or “NIH”) is paying for this research to be done.  Gia: Children's National and a company called Invitae are working together on this research study. | | |
| Patient: Working together - cool! | Patient: What’s Invitae?  Gia: Invitae is a company that provides clinical genetic testing.  Patient: What’s Children’s National? / Thanks for the info!  Gia: Children’s National is a pediatric hospital in Washington, DC.  Patient: Ah, okay | Patient: What’s Children’s National?  Gia: Children’s National is a pediatric hospital in Washington, DC.  Patient: What’s Invitae? / Thanks for the info!  Gia: Invitae is a company that provides clinical genetic testing.  Patient: Got it! |
| Gia: We are recruiting patients from all over the country, with all kinds of backgrounds and experiences with genetic testing. | | |
| Patient: Makes sense. | Patient: How many people will be involved?  Gia: We are hoping to enroll up to 13,000 people.  Gia: We expect half to be patients and family members referred through Children's National, and half to be from outside of Children's National.  Patient: Got it | |
| Gia: It’s really important to understand that research testing like this does not replace clinical testing.  Gia: Whether you join the PMGRC research study or not, you should always continue getting medical advice from your doctor, which might include other options for genetic testing.  Gia: You might want to discuss this study with your family and anyone else you trust before making your decision. | | |
| Patient: I definitely will. | Patient: Who should I talk to?  Gia: Some people you could talk to about your participation in this study include your family members, your doctor, or anyone you trust to help you make decisions  Patient: Okay | |
| Gia: So! Let’s talk about what will actually happen when someone enrolls in this PMGRC study.  Patient: Yes, please!  Gia: At the end of this chat (or after talking to a study team member), if you decide that you do want to join, you will sign an electronic consent form to officially enroll as a participant.  Gia: Each participant will be assigned a unique study ID number.  Gia: This unique study ID will be used to label your study samples and data (instead of using your name or other identifying information).  Patient: That’s reassuring.  Gia: Once enrolled, you will be asked to provide some medical information and detailed family health history.  Gia: The medical information we collect may include things like doctors notes, diagnoses, and past test results that may help us understand how your genetic information affects your health.  Gia: If you had previous genetic testing (such as whole genome sequencing), you may be asked to provide the data from this testing. | | |
| Patient: Sounds easy enough. | Patient: Where do I get my doctor’s notes?  Gia: Most doctors offices and hospitals have a “Medical Records” office or a patient portal where notes and test results can be sent directly to the patient.  Gia: Our study staff can help you take the next steps, if necessary.  Patient: I see / Where do I get my genetic testing data? | Patient: Where do I get my genetic testing data?  Gia: That’s a complicated process, but usually it involves signing a form to send to the lab that did the original testing.  Gia: Our study staff can help you take the next steps, if necessary.  Patient: Okay / Where do I get my doctor’s notes? |
| Gia: Please be aware that the information we collect will not be provided back to you directly.  Patient: Okay  Gia: Once enrolled, you may be asked to provide a tissue sample for genetic testing. This sample could be blood, saliva (spit), or cheek swab (also called “buccal swab”).  Gia: However, not every PMGRC participant will need to provide a sample. | | |
| Patient: I understand. | Patient: Where will my blood be drawn?  Gia: There are a few options.  Gia: If you are having clinical blood drawn for another reason, an extra sample may be collected for this study at that time.  Gia: You could also schedule a blood draw at the Children's National Clinical Research Unit (CRU).  Gia: Or our study team may be able to arrange for a mobile phlebotomy company to come to your house for a blood draw.  Patient: So there are options. Got it. / What’s a buccal swab? | Patient: What’s a buccal swab?  Gia: A “buccal swab” is a large Q-tip used for swabbing the inside of the cheeks.  Gia: If you provide a buccal sample, these will be collected either on site at Children’s National or with a kit provided to you with instructions for collection at home and mailing back.  Patient: Cool! / Where will my blood be drawn? |
| Gia: And that’s pretty much it! There are no other required interactions.  Patient: So what happens next?  Gia: Once you’ve enrolled and provided the requested information and/or samples, a team of study investigators will review your case history and genetic results.  Gia: This team will decide what research tests will be performed on your samples and data. | | |
| Patient: That’s easy! | Patient: What kinds of research tests?  Gia: Your sample may be used for a variety of genetic testing including, but not limited to: Whole Genome Sequencing, Optical Mapping, and RNA sequencing.  Gia: These are different ways of looking at your genetic material. | |
| Gia: Our PMGRC study staff might contact you to request additional samples, more information, or photographs from you.  Patient: What if I don’t want to provide additional samples or photographs?  Gia: You can decline these additional requests at any time and still be part of the study.  Patient: Who has access to the study information?  Gia: As we mentioned before, the samples and data we gather from you will be labeled only with your unique study ID number. This means that your data and samples will be de-identified.  [Tooltip: Data and samples are called “de-identified” when researchers remove any identifiers like your name, address, date of birth, and phone number.]  Gia: Only a few members of the study team will be able to link your study number to your name.  Gia: Other collaborators (such as Invitae or a mobile blood draw company) would only have your name and identifiers if you used that company for previous genetic testing or blood collection.  Patient: That’s good to know.  Gia: Our PMGRC study is one of 5 Genomics Research Centers, and we collaborate with the other 4 centers.  Gia: We might share your genetic information and health information with these other experts, but any information we share will be de-identified, so these centers will not have access to your name or other personal information that directly identifies you.  Gia: This study will send de-identified data to a National Institutes of Health (NIH)-designated data repository.  [Tooltip: A data repository is a place where data are stored for use in future research.]  Gia: This repository may be accessed by other researchers around the world.  Patient: Could my information be used anywhere else?  Gia: The results of this research may be presented at scientific meetings or in scientific publications.  Gia: But you will not be personally identified and no identifiable photos will be published without your separate consent.  Patient: Okay, that’s good to know.  Gia: Your de-identified **genetic information** and **health information** could be used for future research studies or given to another investigator for future research studies without your additional informed consent.  Gia: This de-identified information may be used for many different research studies, for many years in the future.  Gia: If you enroll in the study, you will have the option to let us know if you want to allow your **samples** to be used for other research beyond this study.  Gia: Otherwise, your samples will remain at the Children’s National Research Institute for use in this study only.  Patient: Okay / Good to know!  Gia: So far we’ve been talking about the ways that de-identified information may be used or shared, but you might be wondering whether identifiable information will ever be shared.  Gia: Your identifiable personal information will not be given to anyone else unless you give your permission in writing first, with a few exceptions that I will explain in a moment.  Gia: The study team will be careful with the information we gather for the study, and we will only share it with authorized members of the study team or people who have a need to review this information.  Patient: Who might have a need to review the study information?  Gia: There are some third parties such as government agencies or other groups within Children's National that may check records that identify you without your permission.  Gia: They might review the study records and your medical records to make sure we are following the law and protecting the people in the study and to make sure our study results are correct.  Gia: The agencies or groups who might see these records are:   - the Department of Health and Human Services Office of Human Research Protections - the National Human Genome Research Institute (part of the NIH) - the Children's National Hospital Institutional Review Board (the ethics board that reviewed and approved this research study) - the Office for the Protection of Human Subjects   Patient: Okay, that makes sense  Gia: I can provide you with a link to the Children’s National **Notice of Privacy Practices** at the end of our chat.  Patient: Okay, thanks.  Gia: You may have heard of HIPAA before, but we want to make sure you know the details and how it applies to your participation in this study.  Patient: Isn’t that something I sign at my doctor’s office?  Gia: Yes, you have probably reviewed the HIPAA information at medical visits.  Gia: HIPAA is a privacy law that protects your individually identifiable health information (also called “Protected Health Information” or “PHI”).  [Tooltip: Health Insurance Portability and Accountability Act (HIPAA).]  Gia: The **HIPAA** law also describes how information about you may be used or shared if you are in a research study.  Patient: That makes sense.  Gia: Under HIPAA, you need to sign an agreement in order for researchers to use or share your PHI for research purposes.  Gia: I can provide you with a link to the Children’s National **HIPAA Information Sheet** at the end of our chat, and you can read the complete HIPAA authorization in the consent document.  Patient: 👍  Gia: It is important that you read this carefully and ask a member of the research team to explain anything you do not understand.  Patient: Can you guarantee that my information will be kept private?  Gia: We will make every effort to keep your information private, but no one's privacy can be totally guaranteed.  Gia: To help protect your privacy, the research team has obtained a **Certificate of Confidentiality** from the Department of Health and Human Services (DHHS).  Gia: With this Certificate, the investigators cannot be forced (for example, by a court order or subpoena) to give information that may identify you in any federal, state or local civil or criminal court, or in any administrative, legislative, or other proceedings. | | |
| Patient: Good to know. | Patient: What if I tell people I’m part of this study?  Gia: It is important that you know that a Certificate of Confidentiality does not stop you or a member of your family from voluntarily giving information to others about yourself or about taking part in this research.  Gia: You should also know that if an insurer or employer learns about your participation and you give them permission to receive research information about you, we cannot use the Certificate of Confidentiality to keep your information private from them.  Gia: This means that you must also actively protect your own privacy.  Patient: Got it | |
| Gia: Let’s talk about what types of results this research could find.  Patient: Yes!  Gia: If your samples and/or data are studied as part of this research, there are different types of results that could be returned to you.  Gia: Like we mentioned earlier, you are being referred to this study because you or your family member has a known or suspected genetic condition based on specific health issues.  Patient: Right.  Gia: This PMGRC study will try to find genetic changes that may be related to those issues.  Gia: These results are called **primary findings,** and they often allow the clinical team to make a diagnosis.  Gia: Later in this chat, you will get to choose whether you want to be told about these primary findings  Patient: Great!  Gia: Sometimes the study may find genetic variants that are not related to the patient’s current known health issues.  Gia: These results are called **secondary findings**.  Gia: Some of these secondary findings are considered **medically actionable**, meaning if these genetic changes are known, then you or your healthcare provider can then take action to try and reduce your risk of developing the health problems, or to better treat or manage the health problems.  Patient: What kind of findings are medically actionable?  Gia: Some examples of medically actionable secondary findings may be genetic variants that cause increased risk for future health issues like heart disease or cancer.  Gia: Secondary findings are often related to adult health risks, but it is possible that we could discover a secondary finding that relates to health issues in childhood.  Patient: Okay, I understand.  Gia: Knowing about a medically actionable genetic variant allows doctors to adjust your medical care plan.  Gia: This can include avoiding certain medications or more frequent checkups with your doctor.  Patient: That’s good to know  Gia: The study could also discover secondary findings are **not medically actionable**, meaning they could be related to an increased risk for certain diseases for which there are no known treatments or medical actions.  Patient: I’ll keep that in mind  Gia: It’s important to note that our research team will not be analyzing every sample for secondary findings, but it is possible that we will identify secondary findings in the course of the study.  Patient: What if I don’t want to know about those kinds of health risks?  Gia: That’s perfectly fine.  Gia: If you do decide to participate in the study, you will get to decide if you want to receive any possible research results. And you can decide if you want to receive some types of results and not others.  Patient: Okay / That’s good to know  Gia: Sometimes genetic testing can find unexpected or surprising results that aren’t related to the genetic health issues in your family.  Gia: For example, sometimes genetic testing can reveal that a child's parent is not actually their biological parent or that two parents are related to each other by blood.  Gia: These types of unexpected results are called **incidental findings**.  Patient: Oh, okay  Gia: We do not plan to go looking for these types of incidental findings and we don’t plan to tell you about them.  Gia: But it is possible that you could figure out these incidental results based on other genetic test results we may share with you.    Patient: I understand  Gia: If the study team finds a genetic change that relates to genetic health issues, we plan to tell you about it if you have opted to receive that kind of research result.  Gia: However, because the results from our study are research results (not clinical results), we will not provide you with an official clinical report.  Gia: And you should know that any research findings will need to be **confirmed in a certified clinical lab** before they can be added to your medical record. | | |
| Patient: Who will pay for that testing?  Gia: You or your insurance company will have to pay for those additional services.  Patient: Got it / What does “confirmed in a clinical lab” mean? | Patient: What does “confirmed in a clinical lab” mean?    Gia: Most tests done on samples in research studies are **only for research** and have no clear meaning for health care.  Gia: If we think that there are research results that might have meaning for you or your family, these tests should be re-done by a certified clinical laboratory.  Gia: Results will only be placed in your medical record once they have been clinically confirmed.  Patient: I understand / Who will pay for that testing? | |
| Gia: Just so you know, we expect this PMGRC study to last at least until 2026, but you can choose to stop participating at any time.  Gia: After signing the Consent/Authorization, you can change your mind and either revoke or withdraw your authorization in the future.  Patient: Good to know!  Gia: To revoke the Authorization, you must send a written letter to the Principal Investigator to inform him of your decision.  Seth Berger, MD, PhD  Children's National Research and Innovation Campus  7144 13th Place NW  Washington, DC 20012  Gia: If you revoke this Authorization, researchers may only use and disclose the PHI that was collected for this research study before you revoked the Authorization.  Gia: If you change your mind and withdraw the Authorization, you will not be allowed to participate in the study.  Patient: I understand  Gia: It is important that you know that anyone who undergoes genetic testing in the United States is protected from certain types of discrimination by a United States federal law called the **Genetic Information Nondiscrimination Act (GINA)**.  Patient: What areas are protected?  Gia: Generally, GINA makes it illegal for health insurance companies, group health insurance plans, and most employers to discriminate against you based on your genetic information.  Gia: However, this does not apply to companies with fewer than 15 employees or the United States military. | | |
| Patient: How is health insurance protected?  Gia: Health insurance companies may not request your genetic information that we get from this research.  Gia: Health insurance companies may not use your genetic information when deciding whether to insure you or the amount of money they will charge you.  Patient: I understand / How are employers involved? | Patient: How are employers involved?    Gia: Employers may not use your genetic information that we get from this research when deciding to hire, promote, or fire you.  Patient: I understand / How is health insurance protected? | Patient: I understand |
| Gia: It’s also very important to know that GINA does not protect you against genetic discrimination by companies that sell life insurance, disability insurance, or long-term care insurance. | | |
| Patient: What does that mean?  Gia: These kinds of additional insurance companies (such as life insurance or disability insurance) might ask you to provide your medical history–including genetic testing results–before allowing you to enroll.  Gia: Because the genetic testing being done in this study is for research only, the results will not be part of any official medical record until they are confirmed by a clinical lab.  Gia: Some families may choose to enroll in these kinds of insurances for their child or themselves before getting a clinically validated genetic test result.  Patient: Okay | Patient: Do I need to worry about this?  Gia: Because the genetic testing being done in this study is for research only, the results will not be part of any official medical record until they are confirmed by a clinical lab.  Gia: Some families may choose to enroll in these kinds of insurances for their child or themselves before getting a clinically-validated genetic test result.  Patient: Okay | Patient: Okay |
| Gia: I’d like to take a moment to give you some info about the **risks and possible discomforts** that may occur as part of participating in this study.  Gia: The greatest risks of this study are related to sample collection, the potential for loss of confidentiality of your study information, and possible psychological risks associated with genetic information.  Gia: That may sound like a lot, so I will go through each of these with you.  Patient: Okay, Gia / 👍  Gia: There are some risks related to taking the blood sample, such as discomfort and/or bruising.  Gia: Infection, excess bleeding, clotting, or fainting can happen.  Gia: There may be minimal discomfort in the cheek if a cheek swab sample is obtained.  Patient: Okay, I understand  Gia: The study team will take care to protect the privacy and confidentiality of your study data.  Gia: Because your samples and data will be labeled only with a study ID, your personal information (like your name) will only be known to certain authorized study team members.    Gia: The team will keep your personal and genetic data on a secure drive, and you will be notified immediately if there is a security breach.  Patient: That’s good to know  Gia: However, even though your samples and data will not be labeled with your name or other personal information, your genetic information is unique to you.  Gia: So it is possible that someone could identify you from your genetic information or learn something about you by looking at your genetic information.  Gia: This risk may increase in the future as technologies advance and as more researchers study your genetic information.  Patient: Ah, I see / That is concerning  Gia: These risks may also affect members of your family.  Gia: You may want to discuss your participation in this study with your family and ask the study team about any questions or concerns you may have.  Patient: Okay, I’ll keep that in mind  Gia: There can also be some risks associated with learning about genetic results that may be discovered through this study.  Gia: Genetic testing can sometimes reveal results that you were not expecting or even things you do not want to know.  Gia: It is possible that learning about your genetic results may make you feel distressed or anxious.  Patient: Ah, okay  Gia: Some results may be more certain than others.  Gia: For example, sometimes we may find a “variant of uncertain significance”, which means that we aren’t sure whether the genetic variant is linked to a specific health issue or not.  Gia: Some people may feel anxious or uncomfortable about uncertain results.  Patient: Okay  Gia: And finally, results from this study may sometimes give you information about a genetic health issue that does not have a clear medical treatment.  Gia: Some people may feel distressed or anxious to learn about a genetic health issue or risk without a known treatment.  Gia: It is up to you to decide if you want to receive any possible genetic results from this study, and you can also decide you want to receive some types of results but not others.  Patient: That’s good to know  Gia: We can also talk a little bit about the **possible benefits** of participating in this study.  Gia: You should know that there may be no benefit to you for your participation in this study, and you will not be paid for your participation in this study.  Patient: Okay, I understand  Gia: Some study participants may benefit by getting a new genetic diagnosis or discovering the genetic cause of a known genetic health issue.  Gia: This type of information can be beneficial because it can help that individual understand more about their health, it can give them an idea of health issues to look out for in the future, and it can sometimes give their clinical team some guidance about testing or screening options.  Gia: In rare cases, learning about a genetic result can even help the individual learn about a specific medical therapy or treatment.  Patient: Okay, that makes sense / That would be great  Gia: Unfortunately, for many people who have a genetic health issue, a specific diagnosis or cause may never be found.  Gia: We do not know if the genetic testing approaches used in this study will be able to help diagnose people who do not yet have a diagnosis.  Gia: However, we hope that, through this study, we will gain lots of new knowledge and understanding that will help us find diagnoses for people in the future.  Patient: Okay  Gia: I’m going to give you a bunch of contact information, and you can write it down if you want. ✏️  Gia: If you have any questions about the study or if you get hurt or sick because of something to do with the study, please contact the principal investigator, Dr. Seth Berger, at 202-545-2756.  Gia: In case of a medical emergency, call 911 or go directly to the hospital. Be sure to tell the Emergency Room personnel and your doctor that you are in this study.  Patient: Thanks for this information.  Gia: If you have any questions or concerns about your privacy rights, you may contact the Children's Hospital Privacy Officer at 202-476-6464.  Gia: If you have questions or concerns about your rights as a study participant, you can contact the Children’s National IRB (Office for the Protection of Human Subjects) at 301-565-8447 or you can contact a research advocate by phone (202-476-5586) or by email (RSA@childrensnational.org).  Gia: For urgent matters related to this study, you can contact the research advocate and pediatric ethics program team pager at 202-259-2082.  Patient: Okay  Gia: Whew! We just went through a lot of information together.  Gia: I’ve gone through all the topics I needed to cover. | | |
|  |  |  |
| Gia: Now, it’s up to you to decide if you would like to give your consent for yourself or your child to participate in the PMGRC study. | | |
| Patient: I am ready to consent!  Gia: Okay, great 😀 | Patient: I do not want to consent.  Gia: Okay - that’s just fine.  Gia: If you are interested in participating in the future, you can always contact our study team at [PMGRC@childrensnational.org](mailto:PMGRC@Childrensnational.org)  Patient: Okay, sounds good!  [Chat complete] | Patient: I’m not ready to decide yet / I have more questions before I decide.  [if “I have more questions before I decide”]  Gia: Okay! I can arrange for someone from our study team to contact you directly by phone or email within the next 2-3 weeks.  Gia: Please provide your preferred contact information.  Patient: [Phone and email entry field]  Gia: Great, thanks! You should hear from the team soon.  Gia: If you have questions now, you can contact us at [PMGRC@childrensnational.org](mailto:PMGRC@Childrensnational.org)  Gia: You can also come back here to continue the chat if you have made a decision.  Patient: I think I am ready to consent / I decided not to consent  [if “I’m not ready to decide yet”]  Gia: Okay! Please take all the time you need.  Gia: As you are considering things, you can scroll through our chat to review our discussion any time.  Gia: To come back to the chat, just use the link you used to access the chat.  Patient: Sounds good.  Gia: When you are ready to decide, you can come back and continue this chat.  Patient: I am ready to consent! / I do not want to consent.  [direct to appropriate path if/when returning to chat and selecting an option] |
| Gia: In a moment, I will ask you to make some choices about how you want to participate.  Gia: But first, I need to ask you some questions to make sure you understand the basics of participating in the PMGRC study.  Gia: And don’t worry - if you answer any of these questions incorrectly, I’ll help remind you of the right answer.  Patient: Okay - I’m ready.  Gia: Question 1: Are you required to participate in the PMGRC study? | | |
| Patient: No - it’s voluntary  Gia: That’s right! | Patient: Yes - it’s required  Gia: Actually, that’s not true.  Gia: Participation in the study is completely voluntary.  Patient: Okay, I understand | |
| Gia: Question 2: Is there an alternative to enrolling in the study? | | |
| Patient: Yes - the alternative is to not enroll.  Gia: That’s correct. | Patient: No - the only option is to enroll.  Gia: Actually, that’s not true.  Gia: You have the option to decide NOT to enroll in the study, and this will not impact the medical care you or your family receive.  Patient: I understand | |
| Gia: Question 3: Is there any cost to you or your insurance provider to participate in the study? | | |
| Patient: Yes - I must pay (or have my insurance pay) to participate  Gia: Actually, there will be no charge to you or your insurance for this study.  Gia: Participation in this study is free.  Patient: Okay, good to know | Patient: No - participation is free  Gia: That’s right! | |
| Gia: Question 4: Will you be paid for your participation? | | |
| Patient: No  Gia: That’s correct. | Patient: Yes  Gia: Actually, that’s not correct.  Gia: You will not be paid for your participation in the study.  Patient: Okay, now I see. | |
| Gia: Question 5: Will you benefit from participating in this study? | | |
| Patient: Yes, definitely.  Gia: Actually, that’s not right.  Gia: Though there may be some direct benefits to you, it is also possible that you may not benefit from the study.  Gia: For example, it is possible that you may learn about a genetic result that leads to a diagnosis or a better understanding of your health.  Gia: But you may not get any results from this study or any direct benefit.  Patient: Okay, I understand | Patient: No, definitely not.  Gia: Actually, that’s not right.  Gia: Though we can’t be sure whether you will benefit from participating in the study, it is possible that you will benefit.  Gia: For example, it is possible that you may learn about a genetic result that leads to a diagnosis or a better understanding of your health.  Patient: Okay, I understand | Patient: Maybe, but not necessarily.  Gia: Yep, that’s right.  Gia: Though we can’t be sure whether you will benefit from participating in the study, it is possible that you will benefit. |
| Gia: Question 6: Are there any risks from participating? | | |
| Patient: Yes, there may be risks  Gia: You’re right, there could be some risks. | Patient: There are no possible risks  Gia: Actually, there are some risks of participating in the study.  Gia: The main risks of this study are related to sample collection, the potential for loss of confidentiality of your study information, and possible psychological risks associated with genetic information.  Patient: I understand | |
| Gia: Question 7: What is the purpose of the study? | | |
| Patient: To get as many samples as possible  Gia: That’s actually not correct.  Gia: The goal of the study is to discover the causes of genetic health issues and better understand the role of genetic variants.  Patient: Okay, I understand | Patient: To discover the causes of genetic health issues and better understand the role of genetic variants.  Gia: Yes, that’s correct. | |
| Gia: Question 8: Will you need to provide samples and/or data? | | |
| Patient: Yes, the study team may collect some samples and/or data.  Gia: That’s correct. | Patient: No, no samples or data are ever needed.  Gia: That’s not quite true.  Gia: In fact, the study team may need to gather some samples and data about you.  Gia: If you enroll, our study team will be collecting information such as health history, genetic data, and clinical records for anyone who is enrolled in this study.  Gia: You may also be asked to provide a sample for genetic testing, including a blood sample or a cheek swab.  Patient: Okay, that makes sense. | |
| Gia: Question 9: Will your personal identifiable data be kept confidential? | | |
| Patient: Yes, the study team will work to keep my information confidential.  Gia: That’s right. | Patient: No, any of my information may be shared publicly.  Gia: Actually, that’s not true.  Gia: The study team will take care to protect the privacy and confidentiality of your study data.  Patient: Okay, that’s good to know | |
| Gia: Question 10: If you are injured as part of your study participation, what can you do? | | |
| Patient: Nothing, I have to figure it out myself.  Gia: Actually, that’s not true.  Gia: If you get hurt or sick because of something to do with the study, please call the Principal Investigator, Seth Berger, at 202-545-2756.  Patient: Okay, now I understand. | Patient: I can contact the principal investigator.  Gia: Yes, exactly. | |
| Gia: Thanks for answering these questions! | | |
| [if 10 correct]  Gia: You got 10 out 10 questions right! | [if less than 10 correct]  Gia: It seems like you had a little trouble with these questions.  Gia:I hope my responses helped clarify. Let’s go through them one more time.  [re-present quiz] | |
|  | [if 10 correct]  Gia: You got 10 out 10 questions right! | [if still less than 10 correct]  Gia: To complete your consent, we’d like to connect you to a member of our study team.  Patient: Okay  Gia: Someone from our team will contact you directly by phone or email within the next 2-3 weeks.  Gia: Please provide your preferred contact information.  Patient: [Phone and email entry field]  Gia: Great, thanks!  Gia: You should hear from the team soon.  Gia: If you have questions now, you can contact us at [PMGRC@childrensnational.org](mailto:PMGRC@Childrensnational.org)  [Go to Next steps (document links)] |
| Gia: Now we’re ready to collect your consent for participation in the study.  [for those enrolling “myself”] Gia: We’ll start by capturing your demographic information and enrollment preferences, and then we’ll ask for your electronic signature.  Patient: Okay, sounds good.  [Form: Demographic Information]  First name  Last name  DOB  Email  Phone  Gia: We would like to store your research samples in a biobank for future research, which we will describe in a moment.  Gia: The samples we store could include genetic material (DNA or RNA), skin or other tissue samples, or cells.  Gia: The biobank is maintained by Children's National Research Institute's Center for Genetic Medicine Research.  Gia: Please let us know how you would like your samples to be used.  [Form: Sample Storage and Use]   - Can we store your samples for purposes of this research study? (Necessary for participation in the study) - Y - Can we store your samples in the Children’s National biobank for purposes of unrelated research? Y/N   Gia: You should know that you may change your mind at a later time and request that your sample be destroyed.  Gia: If you change your mind and want to request that your sample be destroyed, you must send a written request to:  Seth Berger, MD, PhD  Children's National Research and Innovation Campus  7144 13th Place NW  Washington, DC 20012  Patient: That’s good to know  Gia: We would like to store personal health information collected from you in this study (such as your name, date of birth, contact information, etc.) in a database for future research.  Gia: The database is maintained by Children's National Research Institute's Center For Genetic Medicine Research.  Gia: Please let us know how you would like your PHI to be stored and used.  [Form: Storage and use of protected health information (PHI)]   - Can we store your PHI in our database for research related to this study? (Necessary for participation in the study) - Y - Can we use your stored PHI to contact you about future research opportunities that are not related to this study? - Y/N?   Gia: You may recall that we discussed the types of genetic research results that may be found in this study.  Gia: You get to decide which types of results you want to learn about, if any.  Gia: Do you remember the different types of results we discussed, like “primary results” and “medically actionable secondary results”? | | |
| Patient: Yes, I remember | Patient: No, can you remind me?  Gia: **Primary findings** are genetic changes that may be related to the specific health issues that have been seen in you or your family members.  Gia: Primary findings often allow the clinical team to make a diagnosis.  Patient: Okay  Gia: Sometimes the study may find genetic variants that are not related to a patient’s current known health issues.  Gia: These are called **secondary findings**.  Gia: Some of these secondary findings are considered **medically actionable**, meaning if these genetic changes are known, then you or your healthcare provider can then take action to try and reduce your risk of developing the health problems, or to better treat or manage the health problems.  Patient: What kind of findings are medically actionable?  Gia: Some examples of medically actionable secondary findings may be genetic variants that cause increased risk for future health issues like heart disease or cancer.  Gia: Secondary findings are often related to adult health risks. However, it is possible that we could discover a secondary finding that relates to health issues in childhood.  Patient: Okay | |
| Gia: Please complete the form below to let us know what types of results you would like to receive:  [Form: Research Result Return Options]   - Do you want to learn about primary results that are found? - Y/N - Do you also want to learn about medically actionable secondary results? - Y/N - Do you want to learn about secondary results that are NOT medically actionable? - Y/N   Gia: Great! You have now entered all of your choices for your enrollment.  Gia: You are ready to provide your signature to consent!  [Form: Consent]  [List of statements around consent:  I am the study participant or I am authorized to act on behalf of the participant.  I have read this consent form or had it read to me.  I have been invited to take part in a research study. I was told why the research is being done and how long my participation in the study is expected to last. I was told about what will happen in the study and if there are any procedures or drugs that are experimental.  I was told that taking part in this research is voluntary. I also was told that I can decide not to take part or stop being in it at any time without any penalty to me or any change to the quality of care I receive at Children's National.  I was told about the risks and possible discomforts of taking part in this research study. I was also informed if there are any possible benefits to me if I am in this study.  I have been given the chance to ask questions about the study, and my questions have been answered. If I have questions later, I can ask one of the people listed in this form.  I agree to take part in this research study.  I will receive a signed copy of this Informed Consent/Parental Permission form to keep.]  Full name  Today’s date  Current time  [I consent]  Gia: Thank you!  [for those also enrolling “my child/children”]  Gia: Now, let’s talk about next steps for enrolling your child or children.  Gia: How many children are you enrolling? | | |
| Patient: One / Two / Three  Gia: Got it!  Gia: We will now collect demographic information and enrollment preferences and get your electronic signature for each child.  Gia: Let's start with the first child. | Patient: Four or more  Gia: Okay, great!  Gia: To enroll four or more children, it would be best for you to talk to a member of our study team.  Gia: Someone from our team will contact you directly by phone or email within the next 2-3 weeks.  Gia: Please provide your preferred contact information.  Patient: [Phone and email entry field]  Gia: Thank you!  Gia: You will hear from the team soon.  Gia: If you have questions now, you can contact us at [PMGRC@childrensnational.org](mailto:PMGRC@Childrensnational.org)  [Go to Next steps (document links)] | |
| Gia: We will now collect demographic information and enrollment preferences for your child, and then we’ll ask for your electronic signature.  Patient: Okay  Gia: Please provide your child's information below.  [Form: Child’s Demographic Information]  First name  Last name  DOB  Email to use for contact regarding this child  Phone to use for contact regarding this child  Gia: We would like to store your child’s samples (such as genetic material including DNA, RNA, or protein, or cells) in a tissue bank for future research as identified below.  Gia: The biobank is maintained by Children's National Research Institute's Center for Genetic Medicine Research.  Gia: Please let us know how you would like your child's samples to be used.  [Form: Child’s Sample Storage and Use]   - Can we store this person’s samples for purposes of this research study? (Necessary for participation in the study) - Y - Can we store this person’s samples in the Children’s National biobank for purposes of unrelated research? Y/N   Gia: You should know that you may change your mind at a later time and request that your child’s sample be destroyed.  Gia: If you change your mind and want to request that your child’s sample be destroyed, you must send a written request to:  Seth Berger, MD, PhD  Children's National Research and Innovation Campus  7144 13th Place NW  Washington, DC 20012  Patient: That’s good to know  Gia: We would like to store personal health information collected in this study (such as your child’s name, date of birth, contact information, etc.) in a database for future research.  Gia: The database is maintained by Children's National Research Institute's Center For Genetic Medicine Research.  Gia: Please let us know how you would like your child’s PHI to be stored and used.  [Form: Storage and use of child’s protected health information (PHI)]   - Can we store this person’s PHI in our database for research related to this study? (Necessary for participation in the study) - Y - Can we use this person’s stored PHI to contact you about other unrelated research studies? - Y/N?   Gia: You may recall that we discussed the types of genetic research results that may be found in this study.  Gia: You get to decide which types of results you want to learn about, if any.  Gia: Do you remember the different types of results we discussed, like “primary results” and “medically actionable secondary results”? | | |
| Patient: Yes, I remember | Patient: No, can you remind me?  Gia: **Primary findings** are genetic changes that may be related to the specific health issues that have been seen in your child or your family members.  Gia: Primary findings often allow the clinical team to make a diagnosis.  Gia: Sometimes the study may find genetic variants that are not related to a patient’s current known health issues.  Gia: These are called **secondary findings**.  Gia: Some of these secondary findings are considered **medically actionable**, meaning if these genetic changes are known, then you or your healthcare provider can then take action to try and reduce your child’s risk of developing the health problems, or to better treat or manage the health problems.  Patient: What kind of findings are medically actionable?  Gia: Some examples of medically actionable secondary findings may be genetic variants that cause increased risk for future health issues like heart disease or cancer.  Gia: Secondary findings are often related to adult health risks. However, it is possible that we could discover a secondary finding that relates to health issues in childhood  Patient: Okay | |
| Gia: Please complete the form below to let us know what types of results you would like to receive for your child.  [Form: Child's Research Result Return Options]   - Do you want to learn about primary results that are found? - Y/N - Do you also want to learn about medically actionable secondary results? - Y/N - Do you want to learn about secondary results that are NOT medically actionable? - Y/N   Gia: Great! You have now entered all of your choices for your child’s enrollment.  Gia: You are ready to provide your signature to give consent for your child!  [Form: Consent for Child]  [List of statements around consent]  Child’s full name  Full name of consenting individual (parent/legal guardian)  Today’s date  Current time  [I consent]  Gia: How old is this child?   - 6 or younger - 7 to 17 - 18+ under my guardianship   Gia: Thanks!  [if enrolling 1 child, proceed to Next steps section]  [if enrolling 2 children]  Gia: Now let’s focus on your second child.  [present child enrollment form section again, focus on second child]  [if enrolling 3 children]  Gia: Now let’s focus on your third child.  [Present child enrollment form section again, focus on third child]  [Next steps]  [for those who noted any children age 7-17 or age 18+ under parental guardianship]  Gia: Since you have a child who is 7 years or older, a member of the study team will need to talk to your child briefly to tell them about the study and confirm their interest in participating.  Gia: A member of the study team will contact you soon to arrange this discussion and finalize your child’s enrollment.  Patient: Okay  [for those who noted child(ren) under age 7 or no children enrolling]  Gia: We are almost done with the consent process. Just a few more things to tell you.  Gia: Below are links to access some of the documents we discussed during our chat:   - Notice of Privacy Practices - <https://childrensnational.org/-/media/cnhs-site/files/about/nppprivacypractices2.pdf> - HIPAA information - <https://www.cdc.gov/phlp/publications/topic/hipaa.html> - GINA resources - <https://www.genome.gov/about-genomics/policy-issues/Genetic-Discrimination#gina>   Patient: Thank you  Gia: After our study staff has finalized the consent paperwork, we will send you a copy of the signed consent documents via a secure email using the email address you provided above.  Gia: You will receive a separate email with a transcript of our chat.  Patient: Okay, great!  Gia: We will also send you an email with a link to an online form where we will collect health information to complete your study enrollment.  Gia: Once you are enrolled, we will be in touch to let you know if we need to collect any samples from you or your child.  Patient: That sounds good.  Gia: Remember, if you have any questions or concerns, you can always reach out to our team at [PMGRC@childrensnational.org](mailto:PMGRC@childrensnational.org).  Gia: Thank you for completing the consent process with me today!  [Chat complete] | | |
| [Go to top of document](#e7m60cxe5zh3) | | |
